# Neutrophil extracellular traps released by CD177^+^ neutrophils aggravated inflammation and neuronal impairment post-SCI

**DOI:** 10.1186/s12964-025-02553-w

**Published:** 2025-12-07

**Authors:** Siming Dai, Ziyang Zheng, Chengxi Zhang, Ao Xue, Hao Liu, Yin Li, Shenyu Wang, Haofei Ni, Jiaxi Li, Siqi Li, Jian Chen, Guoyong Yin, Shujie Zhao

**Affiliations:** 1 Department of Orthopedics, The First Affiliated Hospital of Nanjing Medical University, Nanjing, Jiangsu China; 2 Jiangsu Institute of Functional Reconstruction and Rehabilitation, Nanjing, Jiangsu China; 3 Spinal Cord Disease Research Center, Nanjing Medical University, Nanjing, Jiangsu China; 4 Department of Pathology, Molecular and Cell-Based Medicine, Icahn School of Medicine at Mount Sinai, New York, NY USA; 5 Nash Family Department of Neuroscience, Friedman Brain Institute, Icahn School of Medicine at Mount Sinai, New York, NY USA; 6 Department of Orthopedics, the Second Affiliated Hospital of Xi’an Jiaotong University, Xi’an, China

**Keywords:** Spinal cord injury, CD177, Neutrophils, Macrophages, Microglia, Neuroinflammation

## Abstract

Spinal cord injury (SCI) causes irreversible motor and sensory dysfunction. Initial mechanical injury breaches the blood-spinal cord barrier (BSCB), triggering a rapid influx of neutrophils. However, previous studies on neutrophils after SCI have been relatively limited, the function and heterogeneity of neutrophils need further study. Here, we identified 3 distinct neutrophil subclusters post-SCI using scRNA-seq in SCI mouse models. Additionally, elevated levels of CD177^+^ neutrophils may aggravate inflammation and neuronal impairment based on our data from SCI patients and mice models. Further in vivo and in vitro assays indicate that CD177^+^ neutrophils induce pro-inflammatory polarization of macrophages and microglia via neutrophil extracellular traps (NETs) formation in a peptidyl arginine deiminase 4 (PAD4) and reactive oxygen species (ROS)-dependent manner thus inducing neuronal apoptosis. Additionally, bone marrow transplantation from *Cd177* knockout (KO) to *Cd177* wild-type (WT) mice improved functional recovery post-SCI. These findings elucidate the role of CD177^+^ neutrophils in SCI-related inflammation and highlight their potential as therapeutic targets.

**Supplementary Information:**

The online version contains supplementary material available at 10.1186/s12964-025-02553-w.

## Introduction

 Spinal cord injury (SCI) is a devastating disease which causes irreversible motor and sensory system dysfunction [1]. After initial mechanical impairment, the infiltration of immune cells evokes a sustained inflammation response which exaggerates the damage by causing neuronal apoptosis, demyelination, and glial scar formation [2]. Therefore, therapies targeting immune inflammatory cascades can mitigate tissue damage and benefit the functional recovery of SCI.

After SCI, breakage of blood-spinal cord barrier (BSCB) triggers a rapid and massive influx of neutrophils in the injured areas and evokes the beginning of the immune microenvironment post-SCI [3]. Neutrophils, a polymorphonuclear leukocyte, have long been considered deleterious in injured tissue contexts given their characteristic functional features such as the production of reactive oxygen species (ROS) and release of proinflammatory cytokines [4]. However, previous studies have found that simply depletion of Ly6G/Gr-1 leukocytes worsens the neurological outcome of mice in SCI, indicating that neutrophils may have contract functions in the pathophysiological process of SCI [5]. Since infiltrating neutrophils consist of multiple subgroups, further investigation is required to articulate their heterogeneity.

CD177, also known as neutrophil-specific antigen B1 and polycythemia rubra vera 1, is a cell surface protein specifically expressed on a neutrophil subset [6]. CD177^+^ neutrophils have been reported to produce high levels of ROS and are prone to forming neutrophil extracellular traps (NETs) [7, 8]. NETs are huge web-like structures composed of decondensed chromatin and several cytosolic and granule proteins including histones, neutrophil elastase (NE), myeloperoxidase (MPO), and other antimicrobials [9, 10]. Recent studies point out that CD177^+^ neutrophils may aggravate inflammatory diseases such as inflammatory bowel disease and biliary atresia through NETs formation. However, insufficient research on NETs in SCI studies motivates us to investigate the mechanisms of NET production and their functional consequences in both experimental SCI models and patients.

Increasing evidence has shown that the altered cellular interactions in the immune microenvironment post-SCI are a key factor impeding functional recovery [11–13]. Recent research has indicated that neutrophils may also interact with circulating or resident immune cells, regulatinfg their function and differentiation [14–16]. However, very few studies have focused on the role of neutrophils in SCI models and their detrimental behavior was mainly highlighted as an effect or consequence of other treatments and conditions [17]. Consequently, a better understanding of the complex cellular interactions involving neutrophils in the immune microenvironment post-SCI is expected to have a positive impact on neurological recovery interventions.

In this study, we investigate the heterogeneity of infiltrating neutrophils post-SCI in mice models by scRNA-seq. We demonstrate that a neutrophil subcluster (marked as CD177^+^ neutrophils) differs from the so-called proinflammation neutrophils. Also, CD177^+^ neutrophils were elevated in peripheral blood in SCI patients and correlated with the severity of the injury. Furthermore, our data reveals that CD177^+^ neutrophils could induce the pro-inflammation polarization of macrophages and microglia by forming NETs in a peptidyl arginine deiminase 4 (PAD4) and ROS-dependent way. In addition, transplantation of bone marrow from *Cd177* knockout (KO) mice to *Cd177* wild-type (WT) mice leads to better functional recovery post-SCI. Overall, these data revealed a mechanism through which CD177^+^ neutrophils participate in modulating inflammation response and revealing the potential role of CD177^+^ neutrophils as prognosis and therapeutic targets in SCI.

## Materials and methods

### Mouse models

All animals used in experiments were male. Adult C57BL/6 mice (8 weeks) were purchased from the Animal Core Facility of Nanjing Medical University, Nanjing, China. *Padi4*^-/-^ mice (C57BL/6) were provided by Prof. Xu Wang (Jiangsu University, Zhenjiang, China). *Cd177*^-/-^ and *Cybb*^-/-^ mice were purchased from GemPharmatech Co. All of the mice were housed with no more than five animals per cage under pathogen-free conditions at an ambient temperature of 22–24 °C and humidity of 50–70% with a 12 h–12 h light-dark cycle before use. For the SCI model, a laminectomy was used to expose the spinal cord at T10 after anesthetizing the animal adequately as previously described [18]. Subsequently, a spinal cord impactor (68097, RWD, CA, USA) was used to create injuries by dropping a rod (weighing 5 g) onto the spinal cord from a height of 6.5 cm. A laminectomy (sham-operated) group without SCI damage was used as the control. To inhibit NET formation in vivo, Cl-amidine (20 mg/kg, HY-100574 A, MCE, China, i.p. once daily) and DPI (1 mg/kg, D2926, Sigma-Aldrich, USA, i.p. once daily) was given before surgery. All experimental animal protocols described in this study were approved by the Animal Care Committee of Nanjing Medical University.

### Behavioral assessment and MEP test

The motor function of mouse after SCI was assessed with Basso Mouse Scale (BMS) behavioral analysis, each mouse was trained before. Briefly, at 7, 14, 21, 28 days after surgery, all mice were placed in an open field individually and observed by two independent investigators and ranked based on ankle movement, hind limb joint movements, trunk position and stability, stepping coordination and paw placement. Catwalk gait analysis was also performed to verify the functional recovery. The CatWalk XT system and respective Catwalk XT 10.6 software (Noldus) were used for automated evaluation of footprints, basal motor behavior, and coordination as previously described [19]. To evaluate the recovery of nerve conductional function, an electrophysiological device (NDI-093) as used to assess MEP at 28 days after surgery. The latency and amplitude were recorded and analyzed.

### ScRNA-seq of immune cells after SCI and data analysis

Single-cell suspension from injured spinal cord of mice at 1, 3, 7 days after surgery were prepared as described above. After incubated with Fixable Viability Stain 780 (565388, BD), PerCP-Cy™5.5-conjugated anti-mouse CD45 (550994, BD), BV421-conjugated anti-mouse CD11b (562605, BD) for 30 min at 4 °C, immune cells were isolated by flow cytometry using BD FACSymphony A5 SORP. Then, immune cells (2 × 10^5^ cells/mL) were loaded onto microwell chip using the Singleron Matrix^®^ Single Cell Processing System. Barcoding Beads are subsequently collected from the microwell chip, followed by reverse transcription of the mRNA captured by the Barcoding Beads to generate cDNA. After PCR amplification, the amplified cDNA is then fragmented and ligated with sequencing adapters. The scRNA-seq libraries were constructed according to the protocol of the GEXSCOPE^®^ Single Cell RNA Library Kits (Singleron). Individual libraries were diluted to 4 nM, pooled, and sequenced on Illumina novaseq 6000 with 150 bp paired end reads.

Raw reads from scRNA-seq were processed to generate gene expression matrixes using CeleScope v1.9.0 pipeline. UMI counts and gene counts of each cell were acquired with featureCounts v2.0.1 software, and used to generate expression matrix files for subsequent analysis. Cells were filtered by UMI counts more than 30,000, gene counts less than 200 or more than 5,000, and mitochondrial content more than 20%. The results of ScRNA-seq were uploaded to the Gene Expression Omnibus (GEO) database under accession number GSE240727. To remove batch effect between samples, harmonypy (0.0.5) was used. Seurat v3.1.2 was used for dimension-reduction and clustering and UMAP algorithm was applied to visualize cells in a two-dimensional space. To identify differentially expressed genes (DEGs), the Seurat FindMarkers function based on Wilcox likelihood-ratio test with default parameters was used to select the genes expressed in more than 10% of the cells in a cluster and with an average log (Fold Change) value greater than 0.25 as DEGs. To investigate the potential functions of DEGs, the GO and KEGG analysis were used and pathways with p adj value less than 0.05 were considered as significantly enriched. The cell-cell interaction analysis was performed by CellPhoneDB v2.1.0 based on known receptor–ligand interactions between two cell types/subtypes.

### Isolation and stimulation of neutrophils

To isolate neutrophils from mouse bone marrows, Total bone marrow cells were harvested from tibias and femurs, filtered through a 40-µm cell strainer, and prepared for subsequent magnetically activated cell sorting (MACS). To isolate neutrophils from mouse lesion cores, single cell suspension from injured spinal cord was performed as previously described. Subsequently, a neutrophil isolation kit (Miltenyi Biotec, Gladbach, Germany) was used according to the manufacturer’s instructions after lysis of RBCs to isolate neutrophils from single cell suspension. Neutrophils from human peripheral blood were isolated by density gradient centrifugation using Polymorphprep (AS1114683, Axis-Shield, Dundee, UK) for 40 min at 400 g without brake. Then, cells were suspended in RBC lysis buffer (Biosharp, China) and washed in sterile PBS before use. Neutrophils were cultured at a density of 1 × 106 cells per ml in 1% RPMI1640 for 1 h before stimulated with PMA for 3.5 h at 37 °C. In some assays, to inhibit NET formation, PAD4 inhibitor Cl-amidine (10 µM, HY-100574 A, MCE, China) and ROS inhibitor DPI (25 µM, D2926, Sigma-Aldrich, USA) were added to pre-treat neutrophils. All culture plates and dishes were coated with poly-L-lysine (P4707, Sigma-Aldrich, USA) and washed for 3 times with sterile PBS before use.

### Isolation of CD177^+^ and CD177^-^ neutrophils

To isolate CD177^+^ neutrophils from human peripheral blood, neutrophils were isolated by density gradient centrifugation as shown above. After stained with primary anti-human CD177 antibody (551899, BD) and secondary donkey anti-mouse Alexa Fluor 488, anti-FITC MicroBeads (130-048-701, Miltenyi Biotec, Gladbach, Germany) were further used to isolate CD177 + neutrophils, and the left cells were considered as CD177^-^ neutrophils.

and BV421-conjugated anti-human CD177 (564240, BD). Cells were analyzed by flow cytometry (BD FACSymphony A5 SORP) and data analysis was assessed using the FlowJo software (V10.9.0).

### RNA sequencing and data analysis

Total RNA of macrophages and microglias was extracted after co-cultured with neutrophils isolated from lesion cores of *Cd177* KO or WT mice for 24 h. Total RNA was extracted from the collected samples using the Universal RNA Extraction CZ Kit (RNC643, ONREW) following the manufacturer’s protocol. RNA quantity was measured with a Qubit 4.0 Fluorometer (Invitrogen), and integrity was assessed by denaturing agarose gel electrophoresis. Next, cDNA was generated using VVAHTS^®^ Universal V8 RNA-seq Library Prep Kit for Illumina (NR605-0, Vazyme) and was purified with VAHTS DNA Clean Beads (Vazyme). The purified products were enriched with 12–15 cycles of PCR to create the final cDNA library and then sequenced on the Novaseq 6000 (Illumina) according to the manufacturer’s instructions. Differentially expressed genes (DEGs) with a fold change > 2 and p value < 0.05 were determined using DESeq2(v1.4.5). The RNA-seq results were uploaded to the GEO database with accession number GSE276427 and GSE276429. Gene set enrichment analysis (GSEA) was further performed to interpret the biological significance of pathways.

### Statistical analysis

Data are presented as mean ± SD (standard deviation) and the exact sample size (n) of each experiment is provided in Figure legends. Data with a normal distribution was analyzed using an unpaired two-sided t-test, one-way or two-way analysis of variance (ANOVA) with GraphPad Prism 8 (GraphPad Software, La Jolla, CA, USA). All tests are two-sided and differences between groups were considered statistically significant when P value < 0.05.

### Ethical approval

All procedures performed in studies involving human participants were in accordance with the ethical standards of the institutional and/or national research committee and with the 1964 Helsinki declaration and its later amendments or comparable ethical standards. Also, all applicable institutional and/or national guidelines for the care and use of animals were followed”.

## Main text

### Profiling of infiltrating neutrophils in mice by scRNA-seq after SCI

Early studies have revealed that the breakdown of BSCB triggers a rapid and massive influx of neutrophils in injured areas after SCI which peaked at 1 dpi (days post-injury), which were validated by flow cytometry (Figure. S1 A and B) and immunofluorescence (IF) staining (Figure. S1 C and D) [3, 20]. We also found that neutrophils were widespread in and around injured areas at 1 dpi and 3 dpi (Figure. S1C).

**Fig. 1:**
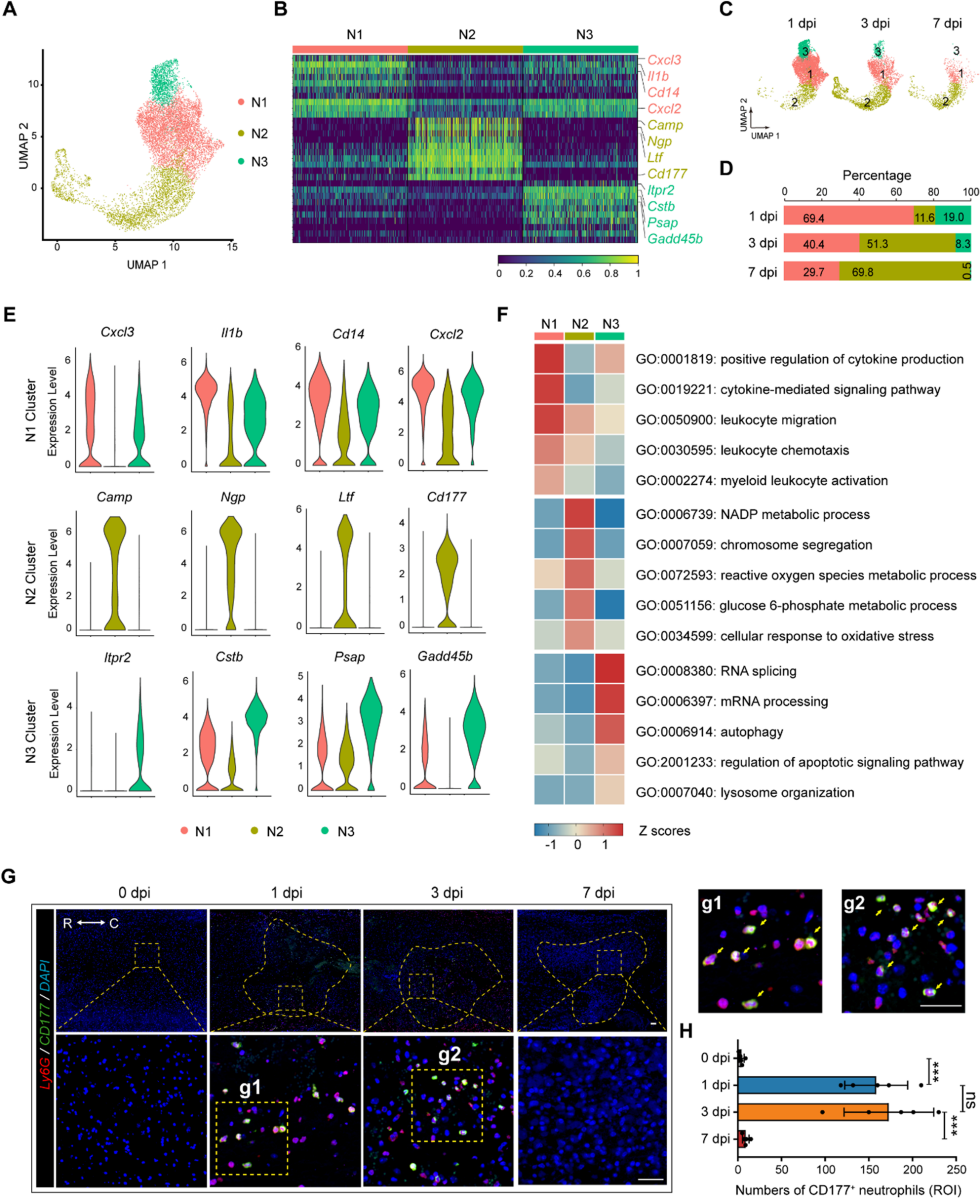
Identification of transcriptomic diversity in infiltrating neutrophils post-SCI at different time points by scRNA-seq.**A** Dimensionality reduction via UMAP of infiltrating neutrophils at 1, 3, and 7 days post-SCI. **B** Differential gene expression analysis showing the top 10 differentially expressed genes (DEGs) for individual clusters. **C** and **D** UMAP (**C**) or bar plot (**D**) showing the proportion of different clusters of neutrophils at varied time points. **E** Violin plot showing marker genes in individual clusters. **F** Representative biography process (BP) categories identified in GO analyses based on upregulated DEGs in each cluster. **G** and **H** Representative IF images (**G**) and quantification (**H**) of CD177^+^ (green) neutrophils infiltrating the injured areas at 1, 3, and 7 days post-SCI (*n* = 5). Scale bar = 200 μm. Dashed lines indicate the lesion center and the boxed areas are shown magnified. R rostral, C caudal. All data are presented as means ± SD; ns indicates not significant, ***P < 0.001; One-way analysis respective p-values indicate of variance (ANOVA) test (**H**)

Growing evidence has shown that neutrophils possess greater functional diversity than previously appreciated and matter in tissue repairment as well [21, 22]. To better understand the heterogeneity of infiltrating neutrophils in mice post-SCI, scRNA-seq was performed to unbiasedly phenotype immune cell transcriptomes (Figure. S2A). After principal component analysis and graph-based cluster analysis (Figure. S2 B-D), we obtained a total of 10,798 neutrophils from 1 dpi (6354 cells), 3 dpi (3272 cells), and 7 dpi (1172 cells) tissue. After comparing gene expression patterns across all neutrophils at all time points, we identified 3 transcriptionally distinct subclusters: N1, N2, and N3 (Fig. 1 A-F). Besides, a combined volcano graph for gene expression in N1-3 cell subpopulations is shown in Figure S3A.

The N1 cluster was the predominant population at 1 dpi (69.4%) and rapidly decreased to 29.7% at 7 dpi (Fig. 1 C and D). This cluster was characterized by strongly expressed genes related to chemotaxis (*Cxcl3*, *Cxcl2*) and inflammation (*Il1b*, *Cd14*) (Fig. 1B, E, and F). This gene expression profile shared similitude with the so-called pro-inflammation phenotype which shows upregulated activity in inflammatory responses, cytokine production, and chemotaxis, indicating a potential role in the recruitment of circulating immune cells and the intensification of inflammatory responses (Figure. 1B, E and F) [23, 24].

The N3 cluster represented a minor subset (19.0%, 8.3%, and 0.5% of all neutrophils at 1, 3, and 7 dpi, respectively) and shared a similar trend as the N1 cluster (Fig. 1 C and D). Highly expression of the lysosome (*Cstb*, *Psap*) and apoptosis (*Itpr2*, *Gadd45b*) genes was observed in this cluster (Fig. 1B, E and F), and KEGG analysis predicted this subpopulation may undergo multiple forms of cell death (Figure. S3B).

We are particularly interested in the N2 subset, which was characterized by increased expression of *Camp*, *Ngp*, *Ltf*, and *Cd177* (Fig. 1B and E). After examining the expression patterns of several top-ranked cluster-specific genes in each cluster, we found that *Cd177* was highly expressed in the N2 cluster (Fig. 1E). Neutrophil subclusters of similar transcriptional features with cluster N2 in our study were observed in several diseases including stroke, myocardial infarction, and biliary atresia, and the elevation of these subpopulations is associated with worse outcomes [7, 21, 25]. GO analysis further indicated increased ROS generation and NADP metabolism in this cluster (Fig. 1 F). Hence, we marked Cluster N2 neutrophils as CD177^+^ neutrophils. Moreover, CD177^+^ neutrophils showed an expanding share in the infiltrating neutrophils post-SCI (from 11.6% at 1 dpi to 69.8% at 7 dpi). Besides, we performed IF staining using antibodies against CD177 and validated the presence of the CD177^+^ neutrophils in vivo at 1,3 and 7 dpi (Fig. 1G and H).

Altogether, our scRNA-seq data revealed three distinct subclusters, and the CD177^+^ neutrophils may participate the pathophysiological process of SCI.

### Elevated circulating CD177^+^ neutrophils predict worse neurological outcomes in SCI patients

**Fig. 2:**
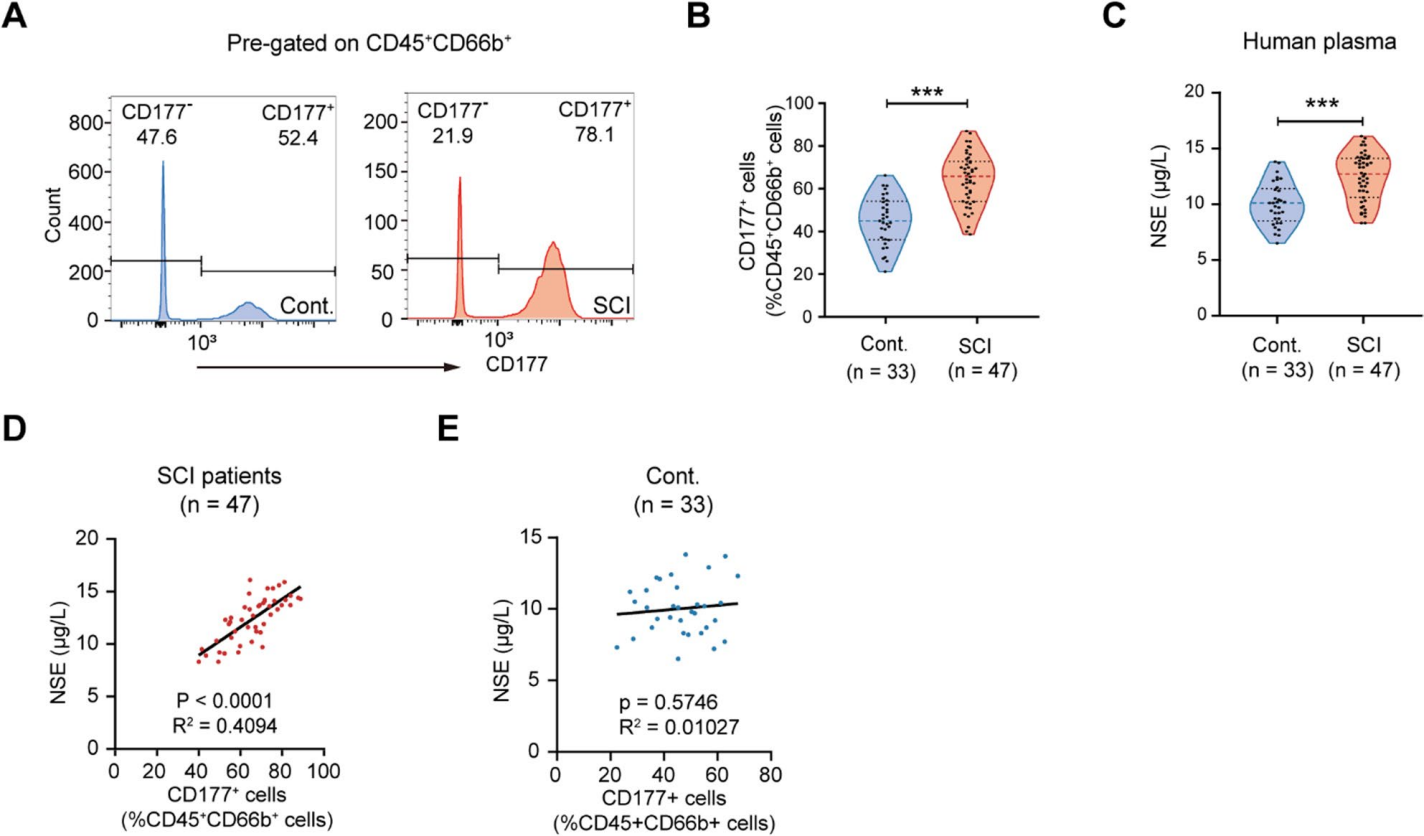
Increased CD177 + neutrophils correlated with exacerbated neurological injury in SCI patients. **A** and **B** Representative flow cytometry analysis (**A**) and quantification (**B**) of the percentage of circulating CD177 + neutrophils from SCI patients (*n* = 47) and healthy donors (*n* = 33). **C** Quantification of NSE levels in plasma from SCI patients (*n* = 33) and healthy donors (*n* = 33). **C** and **D** Correlations of proportions of circulating CD177 + neutrophils with plasma NSE levels in SCI patients (**C**) and healthy donors (**D**). ***P < 0.001; Unpaired two-sided Student’s t-test (**B** and **C**), Pearson correlation coefficient analysis (**C** and **D**)

After uncovering the existence of accumulating CD177^+^ neutrophils in mice post-SCI, we then sought to Figure out whether this trend also existed in SCI patients. We detected the percentage of CD177^+^ neutrophils in the peripheral blood of SCI patients (*n* = 47) and the control group (*n* = 33) using flow cytometry (Figure. S4 A and B). As shown in Fig. 2 A and B, the proportion of CD177^+^ neutrophils of total circulating neutrophils elevated in SCI patients compared with healthy donors, which indicates a potential relationship between CD177^+^ neutrophils and SCI. Additionally, neuron-specific enolase (NSE), a neuronal injury marker, was upregulated in peripheral blood post-SCI (Fig. 2 C). Significantly, peripheral NSE levels correlated with the share of circulating CD177^+^ neutrophils in SCI, while no obvious correlation was observed in the control group (Fig. 2D and E). These results suggested that the accumulation of circulating CD177^+^ neutrophils might connect with the neuronal damage in SCI.

### *Cd177* knockout impaired functional recovery post-SCI

**Fig. 3:**
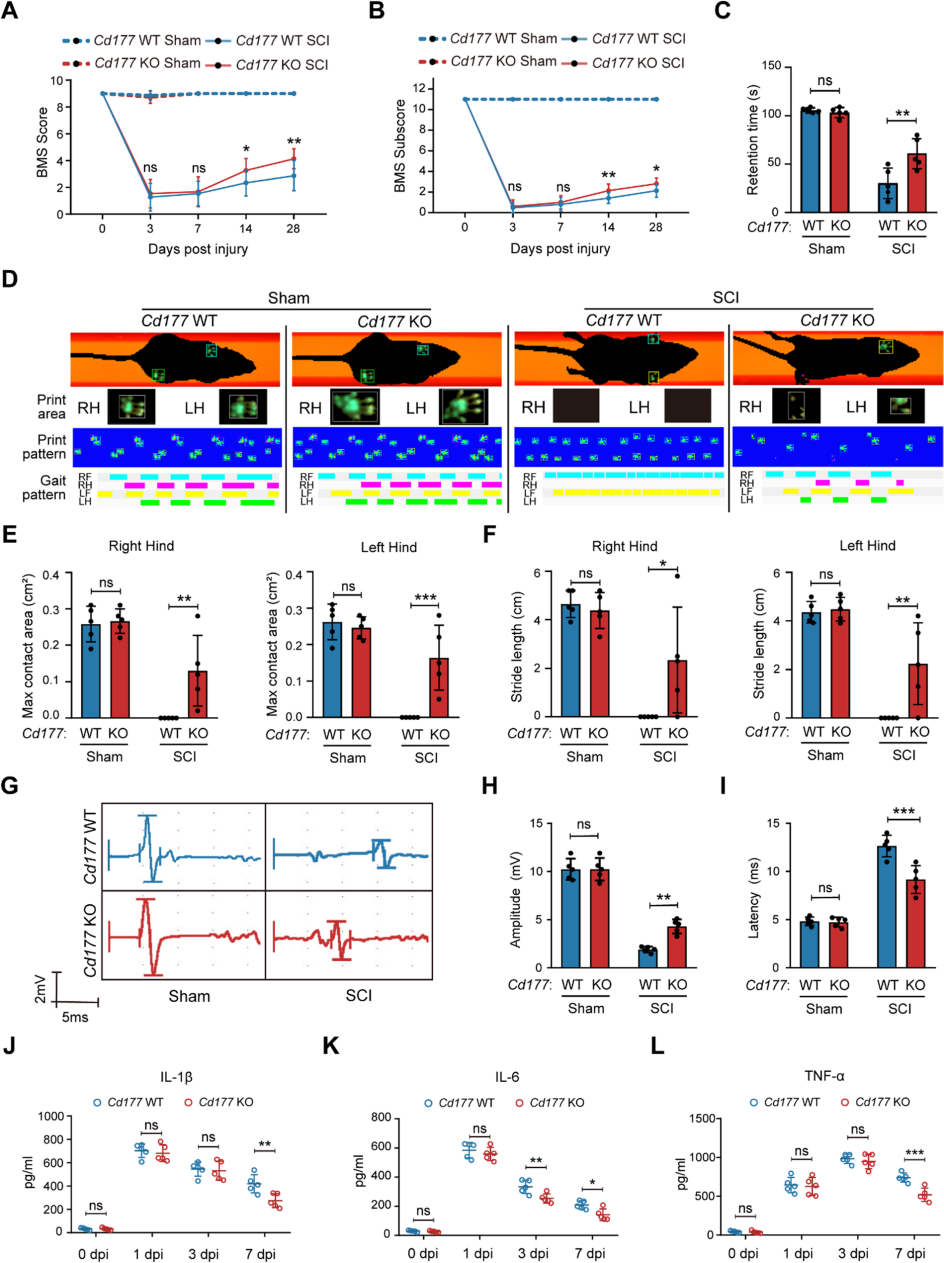
Elevated CD177^+^ neutrophils hindered functional recovery post-SCI. **A** and **B** Statistical analysis of the Basso Mouse Scale (BMS) scores in the Cd177 WT and KO groups over a 28-day period (*n* = 15). **C** Statistical analysis of the rotarod test in the *Cd177* WT and KO groups at 28 dpi (*n* = 5). **D** Representative images showing the maximal print area of left (LH) and right (RH) hind paws, print- and gait pattern (LF = left front paw, RF = right front paw; LH = left hind paw; RH = right hind paw) of Catwalk analysis from the *Cd177* WT and KO groups. Catwalk gait analysis was performed after the final BMS testing at 28 dpi (*n* = 5). **E **and** F** Statistical analysis of Catwalk analysis results (*n* = 5): evaluation of maximal contact paw print area (**E**), stride length of both hind paws (**F**). **G-I** Representative images (**G**) and statistical analysis (**H and I**) of MEP tests of hind paws in the *Cd177* WT and KO groups at 28 dpi (*n* = 5). **J-L** Cytokines related to inflammation in the injured area including IL-1β (**J**), IL-6 (**K**), and TNF-α (**L**) were measured over 7 days post-SCI (*n* = 5). All data are presented as means ± SD; ns indicates not significant, *P < 0.05, **P < 0.01, ***P < 0.001; Two-way repeated measurement ANOVA followed by Turkey post-hoc test (**A** and **B**), One-way ANOVA test (**C**, **E**, **F**, **H** and **I**), or Two-way ANOVA test (**J**-**L**)

The *Cd177* knockout (*Cd177* KO) mice were used to further define the role of CD177 in the functional recovery post-SCI. There was no apparent difference in the open-field locomotion test between the two mouse groups before SCI according to the Basso Mouse Scale (BMS) Scale. In contrast, the functional score of *Cd177* KO mice was notably superior to that of *Cd177* wild-type (*Cd177* WT) mice beginning at 7 dpi (Fig. 3 A and B). Also, *Cd177* KO mice demonstrated better motor coordination and balance abilities with longer retention times in rotarod tests (Fig. 3C). Moreover, gait analysis showed similar results using an automated catwalk gait analysis system. As revealed in Fig. 3D, while *Cd177* WT mice failed to show any hind paw placement after SCI at 28 dpi, the functional restoration of *Cd177* KO mice can be quantified by several parameters such as the area of hind paw footprints. Also, stride length, the base of support (BOS), and the regularity index were measured, indicating a better functional recovery of *Cd177* KO mice (Fig. 3E and F, Figure. S5 J and K). Next, we conducted motor-evoked potentials (MEPs) analysis at 28 dpi to examine the function of descending pathways from the motor cortex to the hindlimb motor neurons. In the *Cd177* KO sham and *Cd177* WT sham groups, similar typical waveforms of MEPs and latency were recorded (Fig. 3G). After SCI surgery, the MEPs were mostly abolished while the *Cd177* KO group presented a larger amplitude and lower latency compared to the *Cd177* WT group, indicating a better restoration of descending motor pathways after genetic disruption of *Cd177* (Fig. 3H and I).

Numerous studies have emphasized the role of neutrophils in the inflammatory response, we hence detected the levels of inflammatory factors in the injured areas after the knockout of *Cd177* [4, 10, 14]. As shown in Fig. 3 J-L, the cytokines levels including IL-1β, IL-6, and TNF-α were significantly decreased in the injured areas of *Cd177* KO mice compared to *Cd177* WT mice at 7 dpi while there was no obvious difference in early timepoints. In line with this, mRNA concentrations of inflammatory cytokines declined in injured areas from *Cd177* KO mice at 7 dpi (Figure. S5 A-C). We then chose 7 dpi as time points to detect inflammation levels based on previous studies and our results. Meanwhile, we measured the number of prime infiltrating immune cells including neutrophils and monocytes by immunohistochemistry and found that *Cd177* knockout did not affect them in number (Figure. S5 D-I). In summary, these results indicated that CD177 hinders the recovery of function post-SCI and may affect inflammatory response post-SCI.

### CD177^+^ neutrophils are essential for SCI-induced NET formation

**Fig. 4:**
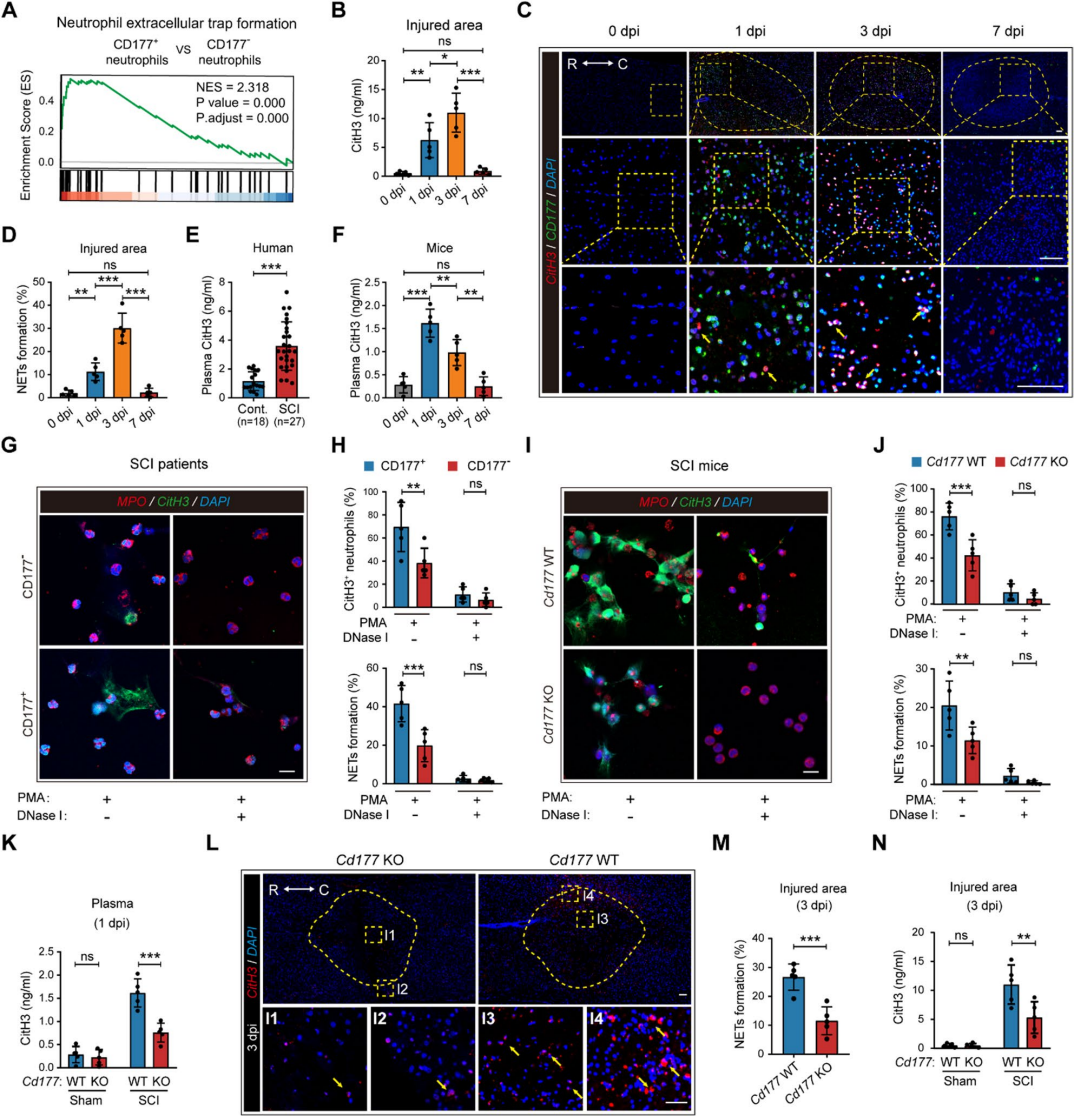
CD177^+^ neutrophils possessed higher NETs formation ability post-SCI. **A** Gene set enrichment analysis (GSEA) showing the distribution of genes in the NETs formation pathway of CD177^+^ and CD177^−^ neutrophils based on the scRNA-seq data. **B** Changes of NET marker CitH3 levels in the injured area of mice were measured over 7 days post-SCI (*n* = 5). **C** and **D** Representative IF images (**C**) of infiltrating CD177^+^ (green) neutrophils with NETs release (red) and quantification (**D**) of NETs formation in the injured areas of mice over 7 days post-SCI (*n* = 5). Scale bar = 200 μm. Dashed lines indicate the lesion center and the boxed areas are shown magnified. R rostral, C caudal. (**E**) Plasma CitH3 levels in healthy donors (*n* = 18) and SCI patients (*n* = 27) were measured and indicated NETs formation in circulation post-SCI. **F** Changes in plasma CitH3 levels of mice were measured over 7 days post-SCI (*n* = 5). **G** and **H** Confocal microscopy images (**G**) of circulating CD177^+^ and CD177^−^ neutrophils from SCI patients stimulated with PMA before treatment of DNase I which degrades NETs and quantification (**H**) of NETs formation in each group. Neutrophils are identified by MPO (red), NETs are labeled by CitH3 (green), and nuclei are stained with DAPI (blue). Scale bar = 20 μm. Results in (**G**) were confirmed in 5 independent experiments using cells from different patients. **I** and **J** Representative confocal microscopy images (**I**) of circulating neutrophils (red) and NETs release (green) from *Cd177* WT or KO mice stimulated with PMA before treatment of DNase I. Scale bar = 20 μm. Quantification of NETs formation in each group was presented in (**J**), *n* = 5. **K** Plasma CitH3 levels of *Cd177* WT and KO mice were measured at 1 dpi (*n* = 5). **L**-**N** The amount of NETs formation in the injured area between *Cd177* WT and KO mice was compared by IF staining (**L** and **M**) of NETs structures (red) and CitH3 levels (**N**) at 3 dpi (*n* = 5). Scale bar = 200 μm. Dashed lines indicate the lesion center and the boxed areas are shown magnified. R rostral, C caudal. All data are presented as means ± SD; ns indicates not significant, *P < 0.05, **P < 0.01, ***P < 0.001; Unpaired two-sided Student’s t-test (**E** and **M**), One-way ANOVA test (**B**, **D**, and **F**), or Two-way ANOVA test (**H**, **J**, **K**, and **N**)

To further identify the function of CD177^+^ neutrophils in the pathophysiological process of SCI, a KEGG pathway analysis was performed based on the scRNA-seq data. As indicated in Figure. S3B, the NET formation pathway was significantly upregulated in CD177^+^ neutrophils. GSEA analysis also validated the enrichment (Fig. 4 A). We then tried to Figure out whether NETs exist in the SCI microenvironment by using a special NET marker CitH3. As displayed in Fig. 4B, the expression of CitH3 in the injured area increased post-SCI and mostly peaked at 3 dpi. Similarly, IF staining validated this trend and revealed that CitH3 was mostly colocalized with CD177^+^ neutrophils, which indicated that CD177^+^ neutrophils may participate in NET formation in SCI (Fig. 4 C and D).

We then tested whether NETs presented in the circulation of SCI patients and SCI mice model. Elevating levels of circulating CitH3 were detected in the plasma of SCI patients at 1 dpi compared to the control group (Fig. 4E). Consistently, plasma CitH3 levels in SCI mice peaked at 1 dpi and gradually returned to normal at 7 dpi (Fig. 4 F). We then isolated circulating neutrophils from SCI mice at 1 dpi and incubated them with or without NETs activator phorbol myristate acetate (PMA) stimulation. As revealed in Figure. S6 A and B, either unstimulated or PMA-stimulated neutrophils from SCI mice showed an obvious increase in CitH3^+^ neutrophils and NET formation, indicating that circulating neutrophils under SCI condition have been primed to undergo NETosis. Similarly, circulating neutrophils isolated from SCI patients also showed higher ability in NET formation compared to the control group (Figure. S6 C and D). To further investigate the connection between elevating circulating CD177^+^ neutrophils and enhanced NETosis in the peripheral blood after SCI, we isolated CD177^+^ and CD177^-^ neutrophils from the peripheral blood of SCI patients at 1 dpi and stimulated with PMA. More NETs formation was observed in CD177^+^ neutrophils and there was no obvious difference in the DNase I treated group (Fig. 4G and H).

We further investigated whether CD177 accounts for the distinctions in SCI-induced NET formation. As revealed in Fig. 4 K, *Cd177* knockout led to decreased plasma CitH3 expression in SCI mice at 1 dpi, while there was no obvious difference in the sham group. Also, compared with the *Cd177* WT group, isolated circulating neutrophils in the *Cd177* KO group showed significantly declined CitH3^+^ neutrophils and NET formation after being stimulated with PMA post-SCI (Fig. 4I and J). Furthermore, we found that genetic disruption of *Cd177* contributes to less NET formation in the injured area (Fig. 4 L and M). In line with this result, levels of CitH3 in the injured area decreased in *Cd177* KO mice compared to *Cd177* WT mice after SCI (Fig. 4 N). In summary, these data revealed that CD177, a receptor on neutrophils, is vital for SCI-induced NET formation.

### SCI-induced CD177^+^ neutrophils’ NETosis is dependent on PAD4 and ROS

**Fig. 5:**
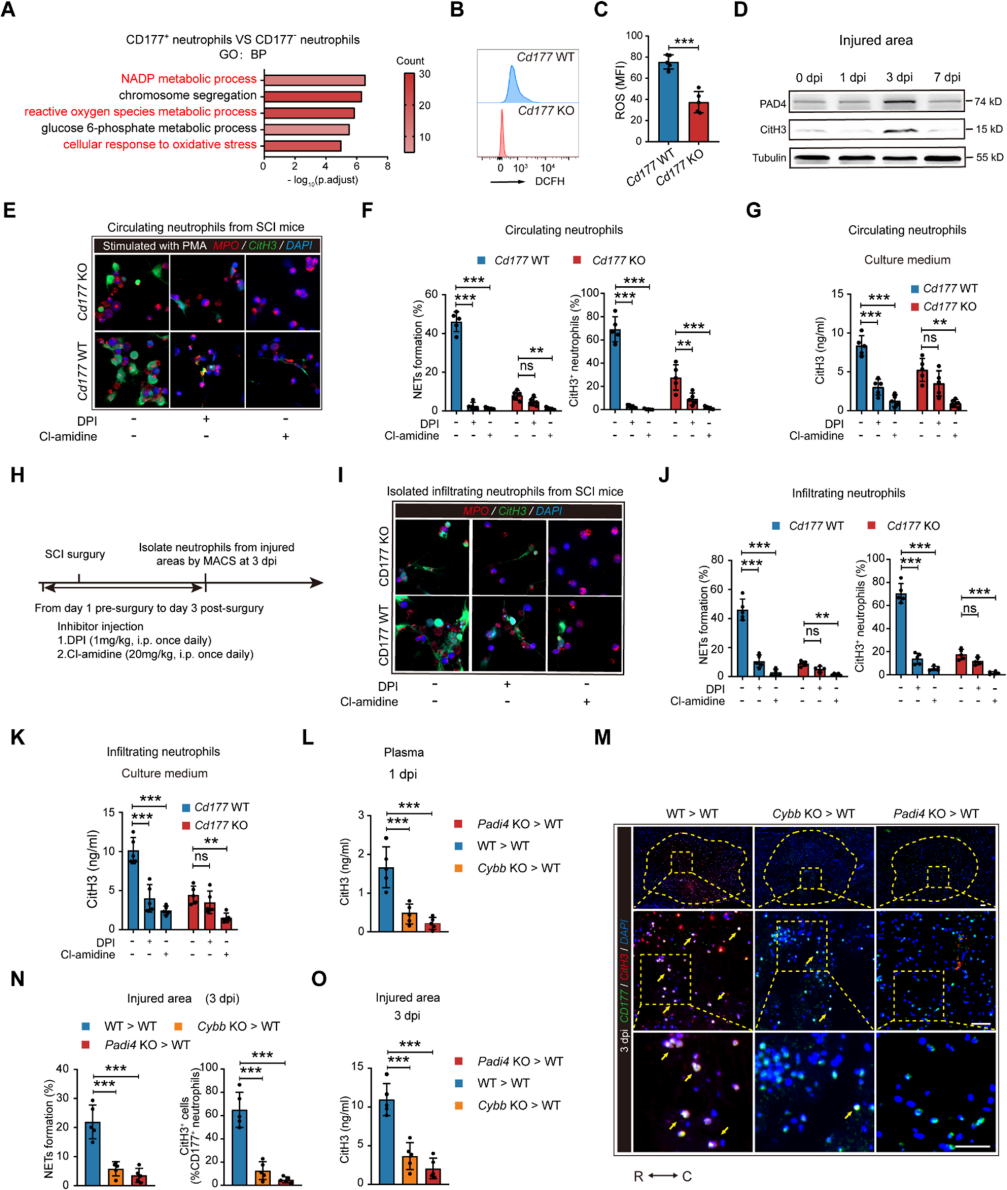
Elevated NETs formation in CD177^+^ neutrophils induced by SCI was dependent on ROS and PAD4. **A** Representative biography process (BP) categories identified in GO analyses based on upregulated DEGs in CD177^+^ and CD177^−^ neutrophils. **B** and **C** Representative histograms (**B**) and statistical analysis (**C**) measuring ROS production in circulating neutrophils from *Cd177* WT and KO mice at 1 dpi (*n* = 5). **D** Changes of PAD4 and CitH3 levels were measured via western blotting over 7 days post-SCI. **E-G** The inhibitory effects of DPI and Cl-amidine on circulating CD177^+^ neutrophils’ NETs formation were confirmed in *Cd177* WT and KO mice at 1 dpi through IF staining (**E**) and detection of CitH3 levels in CM (**G**). Scale bar = 20 μm. Quantification of NETs formation in each group was presented in (**F**), *n* = 5. **H **Experimental design diagram showing inhibitor injection and isolation of infiltrating neutrophils at 3 dpi. **I-K** The inhibitory effects of DPI and Cl-amidine in vivo were tested by detecting NETs formation in infiltrating neutrophils by IF staining (**I** and **J**) and ELISA (**K**). Scale bar = 20 μm, *n* = 5. **L-O** Experimental overview: Bone marrow from WT, *Cybb* KO, and *Padi4* KO mice were transplanted to WT recipients. NETs formation in different groups was measured by IF staining (**M** and **N**) of CD177 (green) and CitH3 (red) double-positive cells in the injured area, CitH3 levels in the plasma (**L**) and lesion (**O**), *n* = 5. Scale bar = 200 μm. Dashed lines indicate the lesion center and the boxed areas are shown magnified. R rostral, C caudal. All data are presented as means ± SD; ns indicates not significant, *P < 0.05, **P < 0.01, ***P < 0.001; Unpaired two-sided Student’s t-test (**C**), One-way ANOVA test (**L**-**O**), or Two-way ANOVA test (**F**, **G**, **J** and **K**)

Next, we intended to elucidate the in-depth mechanism by which CD177^+^ neutrophils undergo NETosis. Several studies have pointed out that NET formations initiate with elevated cytoplasmic ROS generated by NADPH oxidase (mainly nicotinamide adenine dinucleotide phosphate oxidase 2, NOX2/CYBB) or calcium-induced mitochondrial ROS release. Next, granule proteases (mainly MPO and NE) translocation and PAD4-mediated histone citrullination lead to the disassembly of nuclear envelope, chromatin decondensation, and DNA release [9, 26, 27]. However, NETosis triggered by different stimulus can rely on part of the pathways mentioned above [28–30]. Therefore, we detected critical genes related to NET formation based on former studies. As shown in Figure. S3C, *Cybb* and *Padi4* were significantly expressed in CD177^+^ neutrophils. Also, by comparing biological processes (BPs) of CD177^+^ neutrophils with CD177^-^ neutrophils, we found that NADP metabolic process and ROS metabolic process were enhanced in CD177^+^ neutrophils (Fig. 5 A). We then isolated circulating CD177^+^ and CD177^-^ neutrophils from SCI patients at 3 dpi and measured the ROS production using flow cytometry. As displayed in Figure. S6 E and F, CD177^+^ subsets showed increased intracellular ROS levels compared to CD177^-^ subsets. Identically, *Cd177* knockout reduced ROS levels of circulating neutrophils post-SCI (Fig. 5B and C). Moreover, the tissues in the injured area from SCI mice at 3 dpi manifested a significantly increased expression of CitH3 and PAD4 measured by western blotting (WB) (Fig. 5D).

To further investigate the consistency between elevated intracellular ROS production, PAD4 levels, and NETs formation in CD177^+^ neutrophils post-SCI, we pre-treated isolated circulating CD177^+^ neutrophils from SCI patients with Cl-amidine (a PAD4 inhibitor) or DPI (an NADPH oxidase inhibitor) prior to PMA stimulation. All inhibitors decreased NETosis as confirmed by IF staining of NET structures and CitH3 levels in the culture medium evaluated with ELISA (Figure. S6 G-I). Similarly, isolated circulating neutrophils from SCI mice at 1 dpi showed decreased NET formation after inhibitor treatment while *Cd177* knockout led to a decline in inhibitor function, highlighting the critical roles of PAD4 activation and ROS generation in NET formation by CD177^+^ neutrophils (Fig. 5E-G). After investigating the dispensable roles of PAD4 and ROS in SCI-induced CD177^+^ neutrophils’ NETosis, we then estimated the effect of these inhibitors in vivo. We pre-treated mice with Cl-amidine or DPI before SCI surgery and isolated infiltrated CD177^+^ neutrophils in the injured area at 3 dpi for subsequent tests (Fig. 5 H). As revealed in Fig. 5I-K, we observed a significant reduction in both NET markers and NET structures in isolated neutrophils from inhibitor-treated *Cd177* WT mice. However, compared with the *Cd177* WT group, there was no obvious decreased NET formation in the *Cd177* KO group after DPI injection.

Moreover, to confirm the hypothesis that ROS and PAD4-mediated NET formation explains CD177^+^ neutrophils’ NETosis after SCI, we then transplant bone marrow (BM) from WT, *Cybb* KO, or *Padi4* KO mice into WT mice and perform SCI surgery 4 weeks after transplant. In line with our hypothesis, depletion of these genes contributed to decreased NET formation both in circulation and injured areas determined by ELISA analysis and IF staining (Fig. 5 L-O). In summary, these results indicated that SCI-induced CD177^+^ neutrophils’ NETosis relied on a ROS and PAD4-dependent way.

### CD177^+^ neutrophils regulate macrophages and microglia toward a pro-inflammatory phenotype

Next, we sought to explain the way CD177^+^ neutrophils participate in the inflammation response of SCI. As shown in Figure. S7A, no significant enrichment of inflammatory response-associated BPs was observed in the N2 cluster (CD177^+^ neutrophils) compared to the N1 and N3 cluster. We then harvest spinal cords from *Cd177* WT and *Cd177* KO mice at 1 dpi and uncover no obvious distinction of inflammatory genes except for *Il1b* at transcription level (Figure. S7 B). Also, SCI group showed increased release of cytokines while no significant difference was observed between the *Cd177* WT and KO groups (Figure. S7 C-E). These data suggested that CD177^+^ neutrophils themselves are rarely involved in the release of inflammatory factors and there might be other immune cells participating in the inflammatory environment formation.

**Fig. 6:**
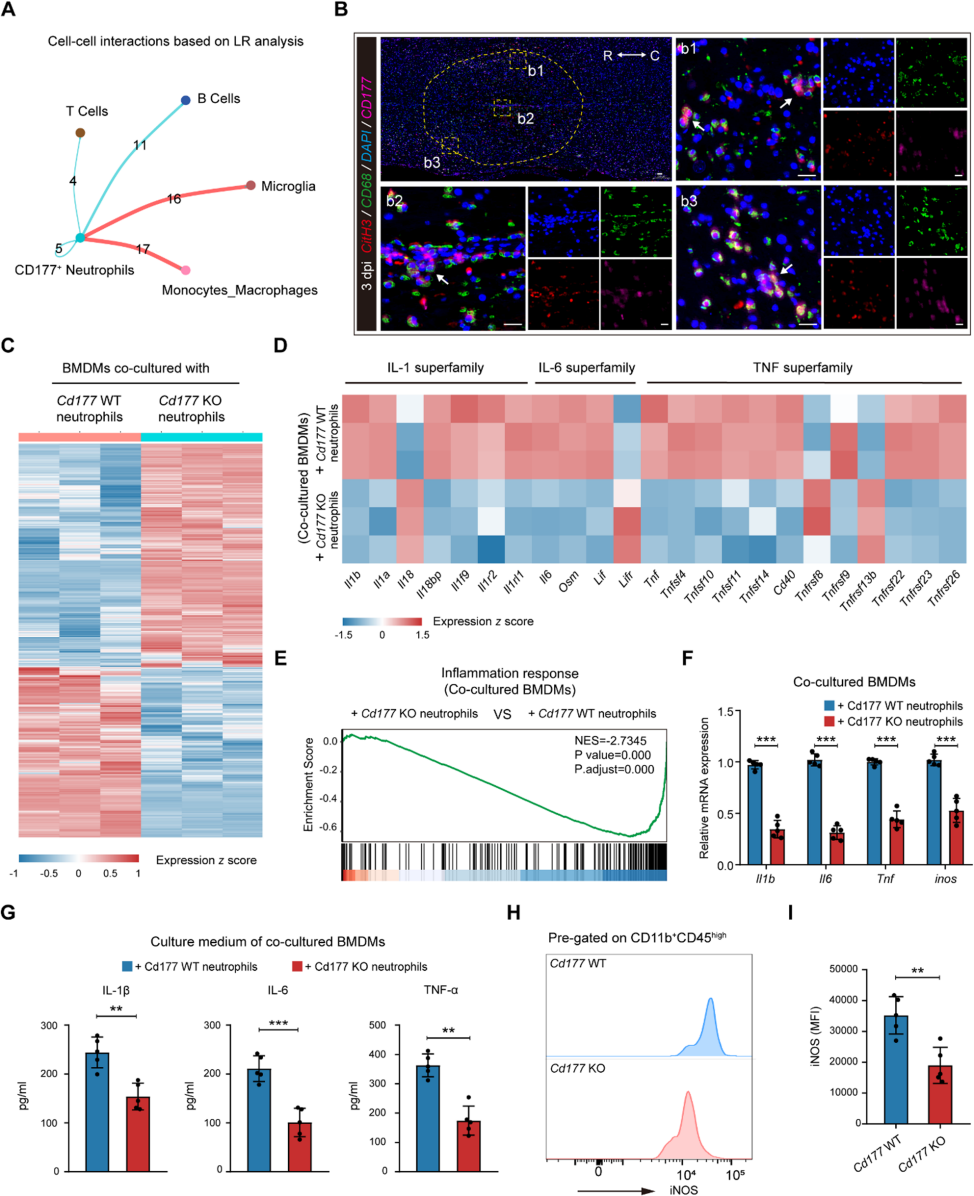
*Cd177* deficiency in neutrophils inhibited pro-inflammatory polarization of macrophages and microglia both in vivo and in vitro. **A** Circle plot depicting ligand-receptor interactions between CD177^+^ neutrophils and other immune cells post-SCI based on the scRNA-seq data. **B** Representative IF images showing the physical proximity of CD177^+^ neutrophils (magenta), CD68^+^ cells (green), and NETs (red) at 3 dpi. Scale bar = 200 μm. Dashed lines indicate the lesion center and the boxed areas are shown magnified. R rostral, C caudal. **C** Heatmap of DEGs in BMDMs co-cultured with isolated infiltrating neutrophils from *Cd177* WT or KO mice at 3 dpi (*n* = 3). Schematic diagram of the co-culture system is shown in Figure. S9A. **D** Heatmap showing DEGs related to cytokine production and release in BMDMs co-cultured with isolated *Cd177* WT or KO neutrophils. **E** GSEA indicating the distribution of genes in the inflammation response pathway based on RNA-seq data. **F** and **G** Quantification of pro-inflammation cytokine levels in the co-culture system measured by qPCR (**F**) and ELISA (**G**), *n* = 5. **H** and **I** Representative flow cytometry analysis (**H**) and quantification (**I**) of iNOS mean fluorescence intensity (MFI) from isolated CD11b^+^CD45^high^ cells (defined as macrophages) from the injured area in *Cd177* WT or KO mice at 7 dpi (*n* = 5). Gating strategy is shown in Figure. S9G. All data are presented as means ± SD; **P < 0.01, ***P < 0.001; Unpaired two-sided Student’s t-test (**F**,** G** and **I**). Data related to microglia are displayed in Figure. S9

Cellular interactions based on the scRNA-seq data were performed and the data revealed macrophages and microglia were closer interaction between CD177^+^ neutrophils (Fig. 6 A). The physical proximity between CD177^+^ neutrophils and macrophages/microglia were then confirmed by IF staining (Fig. 6B). Furthermore, integrated analysis of scRNA-seq data (Figure. S8A) and IF staining (Figure. S8B and C) revealed low *Cd177* expression in macrophages/microglia, with correspondingly absent or minimal protein levels. In addition, LPS stimulation experiments conducted on macrophages/microglia derived from both *Cd177* WT and KO models demonstrated no statistically significant differences in inflammatory responses between the two groups, as illustrated in Figure S8D-G. These findings suggest that CD177 may not directly regulate inflammatory processes in macrophages and microglia. Furthermore, we adopted a direct co-culture system to investigate the influence of CD177^+^ neutrophils on macrophages or microglia post-SCI (Figure. S9A). Neutrophils were isolated in injured lesions at 3 dpi from *Cd177* KO or WT mice and co-cultured with BMDMs or microglia derived from *Cd177* WT mice for 24 h respectively (Fig. 6 C and Figure. S9B). RNA sequencing (RNA-seq) uncovered that compared with their control counterparts, BMDM or microglia co-cultured with isolated *Cd177* KO neutrophils both showed down-regulation of many inflammation response genes (Fig. 6D and Figure. S9C). Additionally, GSEA analysis further verified the above results (Fig. 6E and Figure. S9D). The mRNA expression of *Nos2*, *Il1b*, *Il6*, and *Tnf* were decreased in the *Cd177* KO group compared with the *Cd177* WT group (Fig. 6 F and Figure. S9E). Moreover, compared with the *Cd177* WT group, the levels of inflammation cytokines in the culture medium including IL-1β, IL-6, and TNF-α declined when co-cultured with isolated *Cd177* KO neutrophils (Fig. 6G and Figure. S9F).

After confirming that CD177^+^ neutrophils may regulate the responses of macrophages and microglia towards inflammation in vitro, we then investigated their functions in vivo. Macrophages and microglia were isolated from injured areas using FACS at 7 dpi. The gating strategy is shown in Figure. S9G and iNOS^+^ macrophages shrink in *Cd177* KO mice post-SCI (Fig. 6 H and I). Also, we found that genetic disruption of *Cd177* could decrease iNOS^+^ microglia (Figure. S9 H and I). Collectively, these results suggested that CD177^+^ neutrophils may regulate macrophages and microglia toward a pro-inflammatory phenotype.

### CD177^+^ neutrophils functionally regulated macrophages and microglia via NETs

**Fig. 7:**
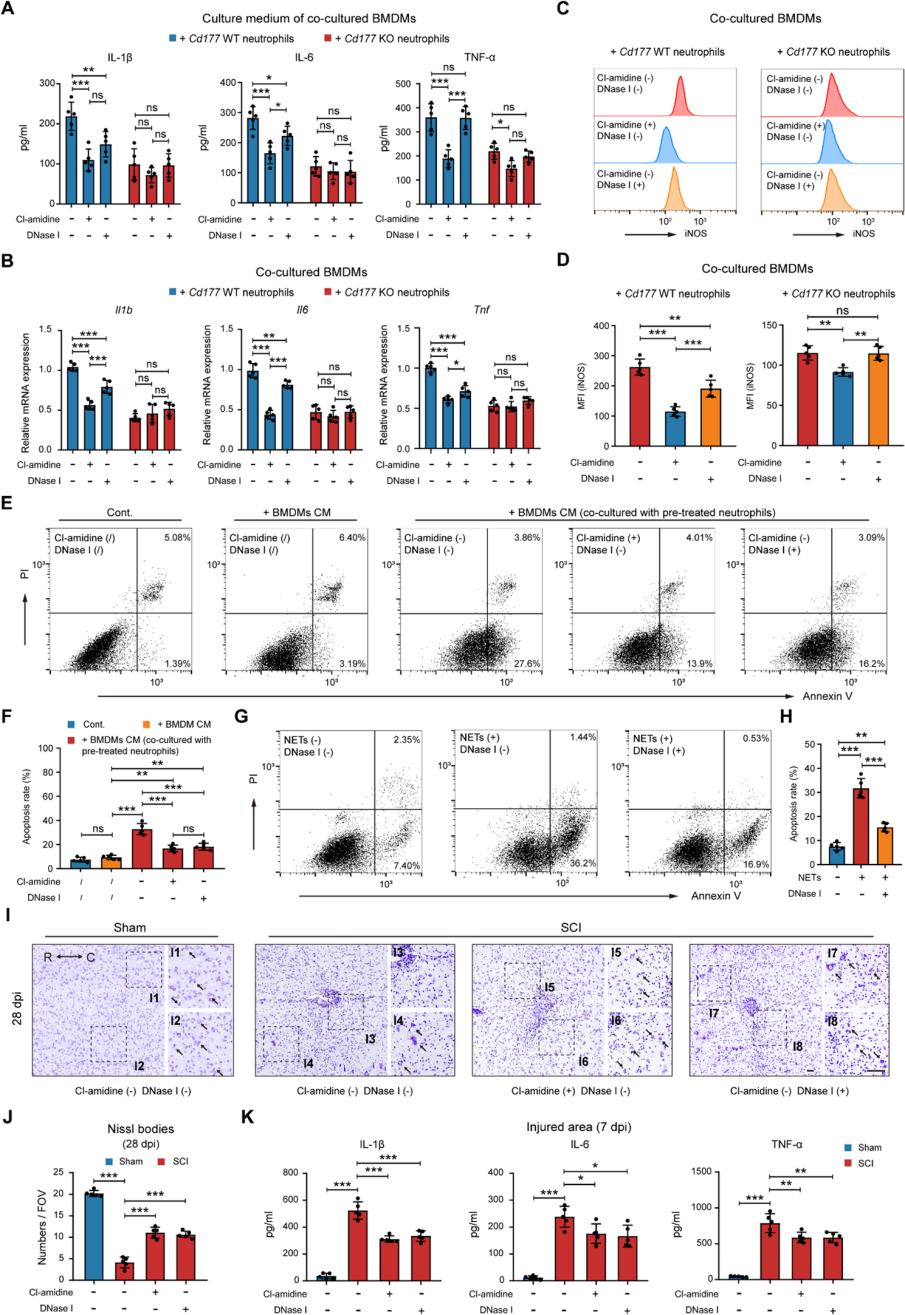
Inhibition of NETs formation attenuates CD177^+^ neutrophil-induced pro-inflammatory polarization of macrophages and microglia thereby reducing neuronal apoptosis. **A** and **B** Relative pro-inflammation cytokines of BMDMs were measured through ELISA (**A**) or qPCR (**B**). BMDMs had been co-cultured with isolated *Cd177* WT or KO neutrophils which were pre-treated with Cl-amidine, DNase I, or vehicle controls (*n* = 5). **C** and **D** Representative histograms (**C**) and statistical analysis (**D**) of the MFI show the expression of iNOS on the BMDMs co-cultured with isolated *Cd177* WT or KO neutrophils which were pre-treated with Cl-amidine, DNase I, or vehicle controls (*n* = 5). **E** and **F** Representative flow cytometry analysis (**E**) and quantification (**F**) of neuronal apoptosis in different groups through Annexin V-FITC/PI double staining. Neurons were treated with vehicle controls, BMDMs CM, or BMDMs CM (co-cultured with pre-treated neutrophils), *n* = 5. **G** and **H** Representative flow cytometry analysis (**G**) and quantification (**H**) of neuronal apoptosis in different groups through Annexin V-FITC/PI double staining. Neurons were treated with vehicle controls, NETs, or NETs with DNase I, *n* = 5. **I** and **J** Representative photomicrographs (**I**) and quantification (**J**) of the Nissl-stained neurons in mice pre-treated with Cl-amidine, DNase I, or vehicle controls post-SCI or sham surgery at 28 dpi (*n* = 5). Scale bar = 200 μm. The boxed areas are shown magnified. R rostral, C caudal. (**K**) Relative pro-inflammation cytokines in the injured area measured by ELISA in mice pre-treated with Cl-amidine, DNase I, or vehicle controls after SCI or sham surgery at 7 dpi (*n* = 5). All data are presented as means ± SD; ns indicates not significant, *P < 0.05, **P < 0.01, ***P < 0.001; Two-way ANOVA test (**A**, **B**, **D**, **F**, **H**, **J** and **K**). Data related to microglia are displayed in Figure. S10

We then investigated the mechanism of macrophages and microglia pro-inflammatory polarization induced by CD177^+^ neutrophils post-SCI. Recently, researchers have indicated the pro-inflammatory role of NETs in several diseases [7, 8, 31]. As shown in Figure. 6B, macrophages/microglia were spatially adjacent to CD177^+^ neutrophils which formed NETs. Next, we sought to figure out whether NETs formed by CD177^+^ neutrophils amplified inflammation response post-SCI. We used Cl-amidine to block NET formation and DNase I to degrade NET structures in the co-culture system mentioned above. As displayed in Fig. 7 A and Figure. S10A, both inhibitors significantly decreased the levels of inflammation cytokines in the culture medium (CM) of *Cd177* WT BMDMs or microglia, while no obvious changes were found in the *Cd177* KO groups. Similar variations were confirmed in the FACS-sorted BMDMs/microglia by polarization marker iNOS (Fig. 7 C-D and Figure. S10 C-D) and qPCR (Fig. 7B and Figure. S10B). Moreover, we found that Cl-amidine tends to be more efficient than DNase I, possibly because simply degrading the DNA scaffold cannot eliminate the influence of specific substances secreted by CD177^+^ neutrophils.

To explore the direct and indirect harmful effects of NETs on neurons, BMDMs/Microglia CM, CM of BMDMs/microglia co-cultured with pre-treated *Cd177* WT neutrophils, and isolated NETs were added to investigate their effects on neurons. As shown in Fig. 7E-F and Figure. S10 E-F, BMDMs/microglia (co-cultured with pre-treated *Cd177* WT neutrophils) CM resulted in extensive apoptosis of neurons, and inhibitors targeting NETs partially alleviated the apoptosis rate. Also, NETs themself can induce apoptosis of neurons, which indicates a potentially direct role in neurological impairment post-SCI (Fig. 7G and H). Moreover, mice pretreated with NET inhibitors possess more Nissl-positive cells and better cell structures in the injured area compared to the control group at 28 dpi (Fig. 7I and J). Also, we observed a marked reduction in the expression of inflammatory cytokines in the injured areas following inhibitor injection (Fig. 7 K). Collectively, our results demonstrated that NETs derived from CD177 + neutrophils are essential for promoting pro-inflammatory polarization of macrophages/microglia and subsequent neurological impairment following SCI.

### Expectations of targeting CD177^+^ neutrophils in SCI treatment

**Fig. 8:**
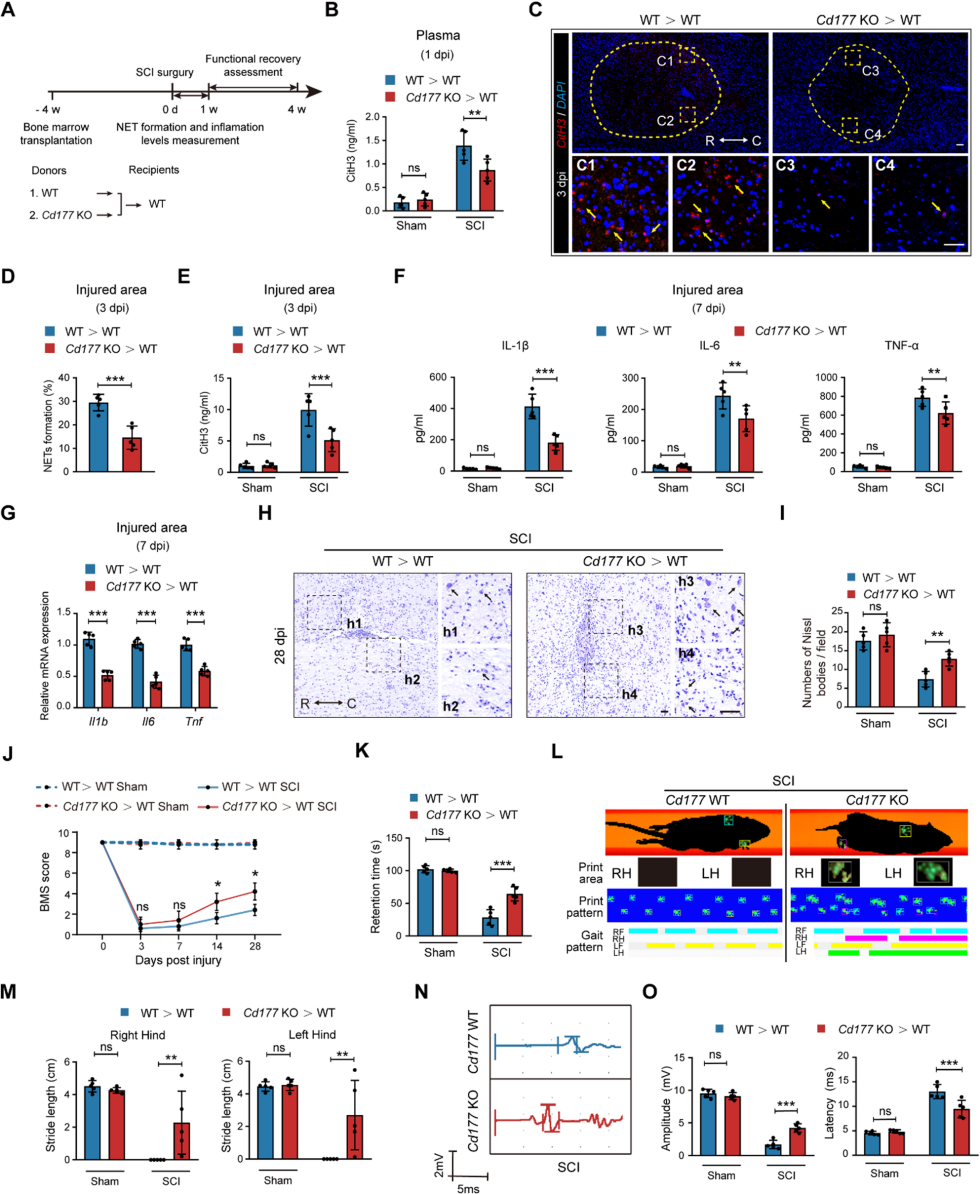
Substitution of *Cd177* WT bone marrow with *Cd177* KO bone marrow eased inflammation and promoted functional recovery post-SCI. **A** Experimental design diagram of Bone marrow transplantation, NET formation and inflammation levels measurement, and functional recovery assessment. **B** Plasma CitH3 levels of *Cd177* KO > WT and WT > WT groups were measured at 1 dpi (*n* = 5). **C-E** The amount of NET formation in the injured area between *Cd177* KO > WT and WT > WT groups was compared by IF staining (**C** and **D**) of NET structures (red) and CitH3 levels (**E**) at 3 dpi (*n* = 5). Scale bar = 200 μm. Dashed lines indicate the lesion center and the boxed areas are shown magnified. R rostral, C caudal. **F** and **G** Relative pro-inflammation cytokines in the injured area were measured through ELISA (**F**) or qPCR (**G**) at 7 dpi (*n* = 5). **H** and **I** Representative photomicrographs (**H**) and quantification (**I**) of the Nissl-stained neurons in *Cd177* KO > WT and WT > WT groups at 28 dpi (*n* = 5). Scale bar = 200 μm. The boxed areas are shown magnified.R rostral, C caudal. (**J**) Statistical analysis of the BMS scores in *Cd177* KO > WT and WT > WT groups over a 28-day period (*n* = 5). **K** Statistical analysis of the rotarod test in *Cd177* KO > WT and WT > WT groups at 28 dpi (*n* = 5). **L** Representative images showing the maximal print area of left and right hind paws, print- and gait pattern of Catwalk analysis from *Cd177* KO > WT and WT > WT groups after SCI surgery. Catwalk gait analysis was performed after the final BMS testing at 28 dpi (*n* = 15). **M** Statistical analysis of Catwalk analysis results (*n* = 5): stride length. **N** and **O** Representative images (**N**) and statistical analysis (**O**) of MEP tests of hind paws in *Cd177* KO > WT and WT > WT groups at 28 dpi (*n* = 5). All data are presented as means ± SD; ns indicates not significant, *P < 0.05, **P < 0.01, ***P < 0.001; Unpaired two-sided Student’s t-test (**D** and **G**), One-way ANOVA test (**B**, **E**, **I**, **K**, **M** and **O**), or Two-way repeated measurement ANOVA followed by Turkey post-hoc test (**J**)

We further explore the therapeutic potential of targeting CD177^+^ neutrophils by using marrow transplantation to explore the effects of treatment targeting CD177^+^ neutrophils (Fig. 8 A). Four weeks after substituting *Cd177* WT BM with *Cd177* KO or *Cd177* WT BM, a standard SCI model was obtained. As shown in Fig. 8B-E, the transplantation of *Cd177* KO BM led to decreased NET formation in circulation and injured areas detected by ELISA and IF staining. Moreover, revised inflammatory response levels were observed after the transplantation of *Cd177* KO BM (Fig. 8 F and G). Nissl staining also revealed an improvement in the number of nissl-positive cells and the integrity of cell structures in *Cd177* WT mice transplanted with *Cd177* KO BM (Fig. 8 H and I, Figure. S11A). We then validated the therapeutic effect on the reconstruction of motor neural function after *Cd177* KO BM transplantation using open-field locomotion tests (Fig. 8 J), rotarod tests (Fig. 8 K), gait analysis (Fig. 8. L and M, Figure. S11 B-E), and electromyography analysis (Fig. 8 N and O, Figure. S11F) as mentioned above. Collectively, these results indicated a promising therapeutic potential for targeting CD177^+^ neutrophils in SCI treatment.

## Discussion

There is emerging evidence indicating the functional role of neutrophil subsets [32–34]. Nevertheless, limited research has specifically addressed the involvement of neutrophils in spinal cord injury (SCI) models, with their adverse effects primarily underscored as a byproduct or outcome of other interventions and conditions. Here, we defined three transcriptionally distinct neutrophil subpopulations in the SCI microenvironment based on our scRNA-seq data to recognize the heterogeneity and distinct functions of infiltrating neutrophils. We found a neutrophil subcluster (marked as CD177^+^ neutrophils) with elevated ROS production and NETs formation abilities. Moreover, a correlation between the increased proportion of circulating CD177^+^ neutrophils and severe neuronal damage was observed in SCI patients. We then sought this panel of neutrophils in different disease models. Accumulating CD177^hi^ neutrophils defined by Gullotta et al. were observed in circulation after stroke in a previous study with a significantly higher ROS production, NADPH oxidase, and NETs-association score compared with other subclusters [21]. The share of CD177^hi^ neutrophils increased in aged mice and resembled worse outcomes compared with young mice. Moreover, a subcluster of neutrophils with similar transcriptional profile (*Cd177*, *Fpr1*, *Mmp8*, *Lcn2*) reported by Ehsan et al. infiltrated 24 h after the onset of myocardial infarction and began to transform into special neutrophil clusters with tissue heterogeneity [25]. Taken together with our findings herein, this supports that CD177^+^ neutrophils not only manifest in various disease models but also harbor the potential to serve as predictive and therapeutic indicators.

Recently, NETs have emerged as mediators for cellular interactions in several diseases, highlighting the importance of elucidating the mechanism responsible for NET formation [9, 35]. Due to the heterogeneity of stimuli and the microenvironments, the molecular mechanisms and composition of NET formation vary from disease. Our discovery indicates that NETs are primarily formed by CD177^+^ neutrophils through a pathway dependent on cellular ROS and PAD4 post-SCI. Similarly, recent research uncovered that CD177^+^ neutrophils, in the biliary atresia (BA) model, can induce cholangiocyte death through the production of NETs [7]. However, in contrast, CD177^+^ neutrophils in the BA model exhibit a higher expression of interferon-stimulated and neutrophil degranulation genes and mediate NET formation through elevated ROS via the mitochondrial pathway, rather than the anticipated NOX2 pathway in our study. This could be attributed to the distinct microenvironments associated with infectious and acute traumatic diseases.

Several studies have indicated the crucial role of macrophages in the formation of inflammatory cascades post-SCI [36–38]. Macrophages, including resident microglia and infiltrating bone marrow-derived macrophages, may take on a proinflammatory state where they possess higher phagocyte abilities and elevated chemokine production [2, 12, 18]. Although interactions between neutrophils and macrophages have been explored in various disease models such as stroke and myocardial infarction, related research in SCI is not yet sufficiently comprehensive [35, 39]. We have observed for the first time that in the SCI microenvironment, CD177^+^ neutrophils can regulate macrophage towards a pro-inflammatory phenotype through NETs both in vivo and in vitro. Our findings partially explain the mechanism by which neutrophils are involved in regulating the sustained inflammatory response post-SCI, and the specific molecular mechanisms involved warrant further research.

However, there remain some limitations of this study that require further research. Given the limited accessibility to infiltrating neutrophils in human SCI lesions, we focused on peripheral blood as a feasible alternative, and cross-validated the findings in a mouse model. However, we did not perform single-cell RNA sequencing of peripheral blood in mice. It is also important to note that the neutrophil subsets circulating in blood may not perfectly mirror those that infiltrate the injury site, given the distinct microenvironment of the SCI. This aspect may require further investigation to clarify potential phenotypic transitions during neutrophil migration. Furthermore, this study lacked scRNA-seq data on infiltrating neutrophils from *Cd177* KO versus WT mice post-SCI. Therefore, whether *Cd177* knockout alters neutrophil subset characteristics and composition remains to be established. Our data lead us to hypothesize that *Cd177* loss reduces the proportion of the N2-like subset, thereby potentially attenuating the overall inflammatory response—a hypothesis that merits further study. Besides, we have yet to elucidate the specific stimuli triggering the formation of NETs by CD177^+^ neutrophils post-SCI, as well as the precise constituents within NETs. Moreover, we incorporated a total of 47 SCI patients in this study, while to test the accuracy and value of CD177^+^ neutrophils as a predictive indicator post-SCI, a multicenter clinical study with a larger patient cohort will be needed in the future to validate these findings. The cross-talk between the neutrophils and macrophages (BMDM and Microglia) is complex, and other molecular components/pathways affected by CD177 need further investigation.

## Conclusions

**Fig. 9:**
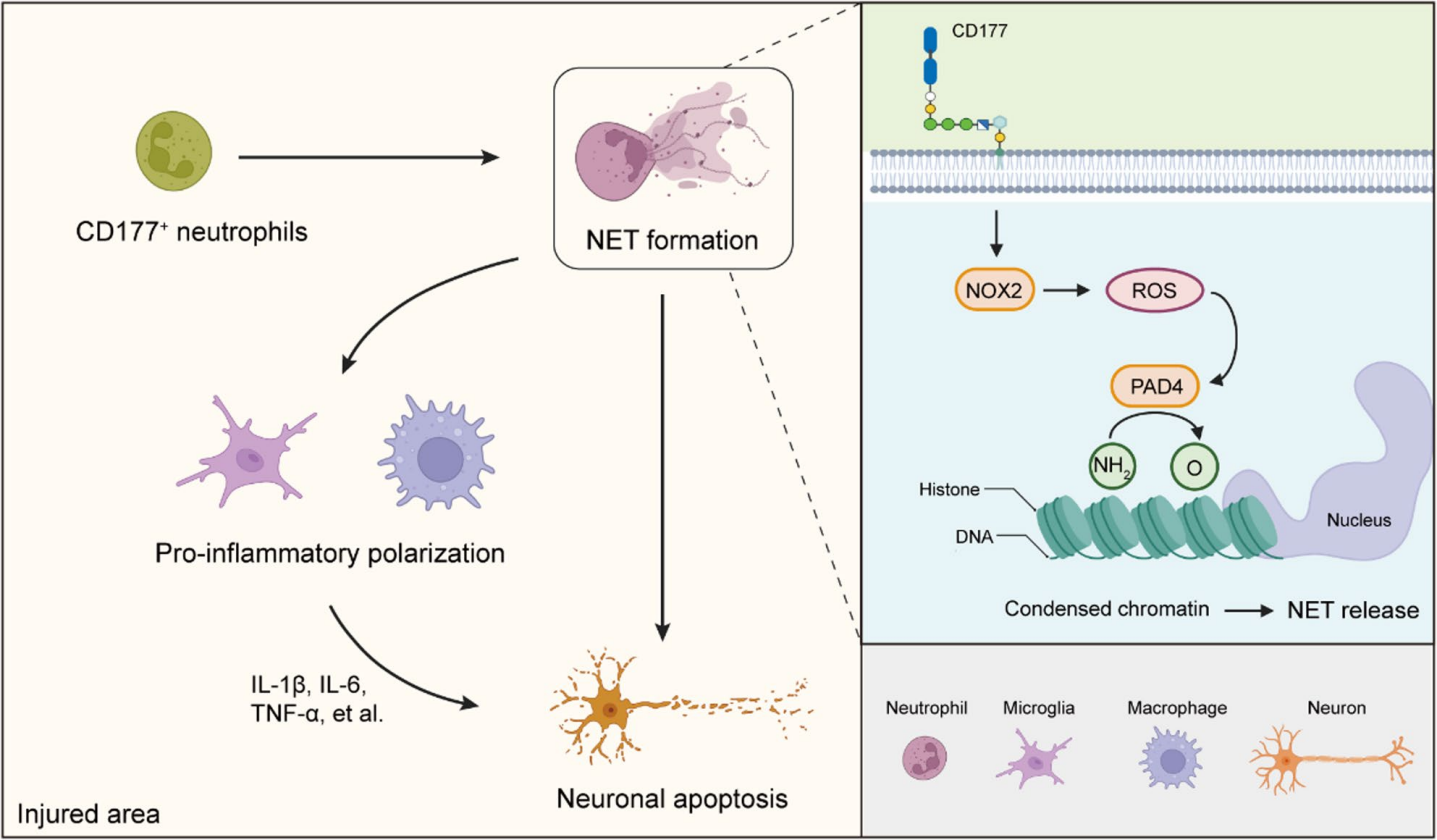
Schematic representation of the potential function and potential regulatory mechanisms of CD177^+^ neutrophils post-SCI. CD177^+^ neutrophils could induce the pro-inflammation polarization of macrophages and microglia by forming NETs in a PAD4 and ROS-dependent way. In addition, NETs themselves can induce apoptosis of neurons. Collectively, these findings elucidate a mechanism by which CD177^+^ neutrophils participate in modulating inflammation response post-SCI

Overall, our study demonstrates the mechanisms of CD177^+^ neutrophils in modulating the SCI microenvironment. Elevated CD177^+^ neutrophils post-SCI could induce the pro-inflammation polarization of macrophages and microglia via NETs, which then contribute to prolonged and exaggerated inflammatory responses (Fig. 9). Thus, neutrophils and NETs may represent a viable target for SCI treatment, and CD177 expressed in neutrophils can be targeted as a therapeutic approach for enhancing functional recovery post-SCI.

## Supplementary Information

Supplementary Material 1.

Supplementary Material 2.

## Data Availability

The datasets generated during the current study are available in the GEO database, GSE240727, GSE276427 and GSE276429.
