# Supplementary material for: Neutrophil extracellular traps released by CD177+ neutrophils aggravated inflammation and neuronal impairment post-SCI
Source: Cell Commun Signal. 2025 Dec 7;24:22. doi: 10.1186/s12964-025-02553-w (PMC12797700; doi:10.1186/s12964-025-02553-w)

Original Images of Representative Western blot images. Related to Fig. 4.

Original Western blots for Figure 4g

a

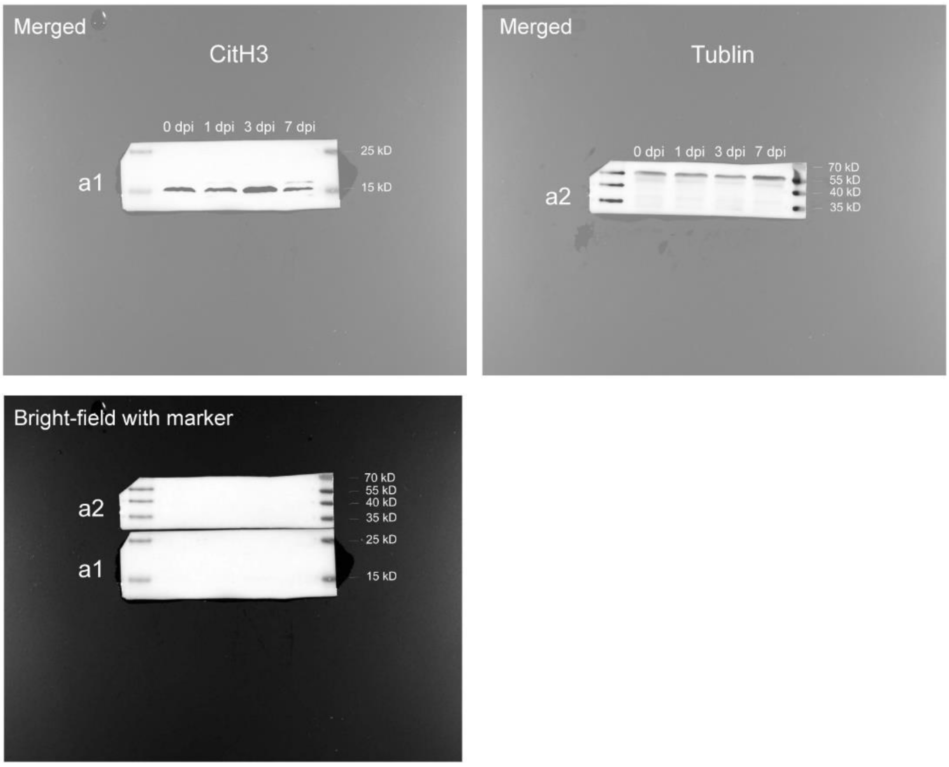

b

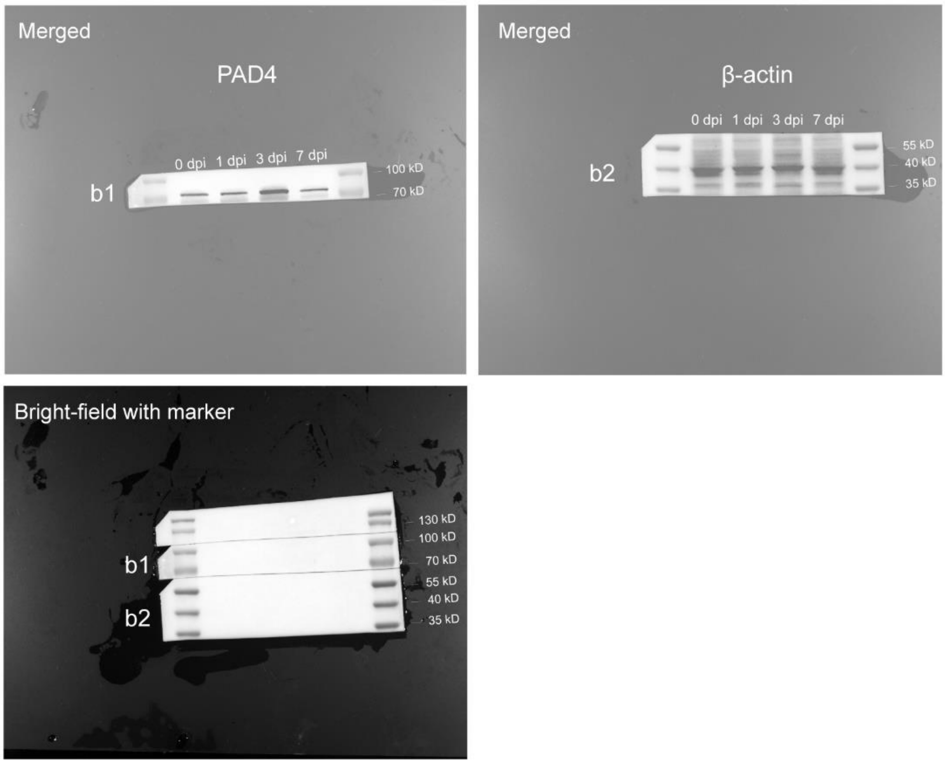

Supplement: Supplementary file 1 — Supplementary Material 1. [file 12964_2025_2553_MOESM1_ESM.pdf]
