# Supplementary material for: Neutrophil extracellular traps released by CD177+ neutrophils aggravated inflammation and neuronal impairment post-SCI
Source: Cell Commun Signal. 2025 Dec 7;24:22. doi: 10.1186/s12964-025-02553-w (PMC12797700; doi:10.1186/s12964-025-02553-w)
Supplement: Supplementary file 2 — Supplementary Material 2. [file 12964_2025_2553_MOESM2_ESM.docx]

**Supplementary materials**

**Supplementary methods**

**Patient sample collection**

This study was approved by the Ethical Committee of The First Affiliated Hospital of Nanjing Medical University, Nanjing, China (2019-SR-017). All biological samples from patients and healthy donors were collected after obtaining informed consent from all participants. A total of 47 tSCI patients admitted to the Department of Spine Surgery, the First Affiliated Hospital of Nanjing Medical University, from December 2020 to June 2022 were included in the present study. Patients with infectious, neoplastic and autoimmune diseases were excluded. All cases received a diagnosis of SCI by combining clinical symptoms (ISNCSCI and ASIA score), electrophysiology, X-ray, and MRI analysis. Peripheral blood samples were collected at 1 dpi before the surgery. All plasma samples were stored at -80 ℃ immediately after centrifugation for 10 min at 3000 rpm. Detailed clinical characteristics are summarized in Table S1 and S2.

**Tissue processing**

At 1, 3 or 7 days after surgery, mice were anesthetized as described above for blood collection and then sacrificed by rapid cervical dislocation. The arterial system was rinsed by perfusion with phosphate-buffered saline from the left ventricle of the heart. For tissue immunofluorescence, the spinal cord was harvested and fixed in 4 % paraformaldehyde and embedded in paraffin. For single-cell suspension preparation, the fresh tissues were minced into small pieces after washed for 3 times with sterile HBSS and then digested with 1ml sCelLiveTM Tissue Dissociation Solution (Singleron) by Singleron PythoN™ Tissue Dissociation System at 37 °C for 35 min. After filtered by 40-micron sterile strainers and lysis of RBCs, the cell suspension was centrifuged and then suspended softly with PBS. For lesion lysate preparation, tissues around lesion core were harvested and washed with cold PBS for 3 times. Subsequently, the specimens were cut into pieces and sonicated. After centrifugation, the supernatants were stored at -80 °C immediately for ELISA test. Additional samples were stored at -80 °C.

**Bone marrow transplant (BMT)**

For performing BMT, the recipient mice (8 weeks) were irradiated at 700 cGray using an X-ray orthovoltage source (RS 2000 Pro, RADSOURCE, USA) as previously reported. Donor bone marrow cells (5 × 10^6^ cells) obtained from WT and CD177^−/−^ donor mice (8 weeks) were intravenously injected into the recipient mice (after irradiation). Four weeks after BMT, SCI surgery was carried out on recipient mice.

**ROS evaluation**

The intercellular ROS levels were measured using the ROS Assay kit (S0033, Beyotime) according to the manufacturer's instructions. Briefly, the cells were collected and incubated with 10 uM DCFH-DA for 20 min in a final volume of 500 ul. Then, the cells were washed three times with serum-free medium and transferred to polypropylene FACS tubes. To summarize, 1 × 10⁵ cells were harvested from each group via FACS, and group differences were evaluated using mean fluorescence intensity (MFI) values for quantification. Data were analyzed using FlowJo.

**Primary cell culture**

Primary BMDMs were isolated and cultured as previously described. Briefly, total bone marrow cells were collected from tibias and femurs as mentioned above. After RBC lysis and centrifugation, cells were resuspended in complete RPMI-1640 medium containing 25 ng/ml macrophage colony-stimulating factor (416-ML, R&D Systems, Minneapolis, MN, USA) and then cultured for 7 days. Primary neurons were isolated from embryonic mice using a primary neuron isolation kit (88280, Thermo Fisher Scientific, MA, USA) according to the instructions of the manual. After centrifugation and washing, neurons were cultured in Neurobasal™ Plus Medium (A3582901, Thermo Fisher Scientific, MA, USA) for 7 days before further tests.

To isolate primary microglia, C57BL/6 neonatal mice were used in this study. The whole brain was carefully extracted and minced into1 mm³ pieces. The minced tissue was then incubated in a mixed enzymatic solution containing 0.25% trypsin and DNase at 37°C for 20 minutes. Following centrifugation and washing, the cell suspension was transferred to a T75 culture flask coated with PLL for culture. Half of the culture medium was replaced every two days. On the 14th day, the culture flasks were placed on an orbital shaker at 37°C and agitated at 180 rpm for 4-6 hours to facilitate cell detachment. The detached cells were collected by centrifugation for further use.

**Histology and immunofluorescence**

For tissue immunofluorescence staining, paraffin-embedded mouse spinal cord sections were blocked with 10% bovine serum albumin (BSA) and then incubated overnight at 4 °C with the following primary antibodies: anti-Ly6G (1:200, GB11229, Servicebio, Wuhan, China), anti-CD68 (1:200, ab283654, Abcam), anti-CD177 (1:200, bs-1482R, Bioss, Beijing, China), anti-CitH3 (1:200, ab5103, Abcam). This was followed using a Tyramide signal amplification (TSA) Staining Kit (G1236, Servicebio, Wuhan, China). Finally, slides were visualized using an Olympus microscope. For cell immunofluorescence staining, the cells were fixed in 4% paraformaldehyde for 30 min, then permeabilized with 0.05% Triton X-100, and finally blocked with 5% BSA. Primary antibodies including anti-MPO (1:200, AF3667, R&D Systems) and anti-CitH3 (1:1000, ab5103, Abcam) were added and the cells were incubated overnight at 4 °C. This was followed by incubation with the following secondary antibodies: donkey anti-mouse Alexa Fluor 488 (1:1000), and donkey anti-goat Alexa Fluor 546 (1:1000). 4′,6-diamidino-2-phenylindole (DAPI) was used to detect DNA, and microphotographs were obtained by a confocal microscope (LSM710, Zeiss, Heidenheim, Germany).

**Western blots**

Mouse spinal cords were lysed using RIPA lysis buffer (89901, Thermo) containing phosphatase and protease Inhibitor (78440, Thermo) according to the manufacture’s instructions. Further, equal amounts (20-40 μg) of proteins were separated by SDS/PAGE gel and transferred to a polyvinylidene fluoride (PVDF) membrane. After blocking with 5% skimmed milk for 1h, the membrane was incubated with the following primary antibodies: anti-PAD4, anti-CD177, anti-CitH3, anti-tubulin at 4 °C overnight followed by species-matched secondary antibodies. The signals were detected by ECL reagents (34578, Thermo, USA). Bands were imaged by ChemiDoc XRS+ Imaging system (Bio-Rad) and assessed using imageJ. Results are expressed as fold changes relative to the internal control.

**ELISA**

To detect NETs activity in plasma and lesion cores, a Citrullinated Histone H3 (Clone 11D3) ELISA Kit (501620, Cayman, Germany) was used according to the manufacturer’s instructions. To detect inflammatory factors activity in cell culture supernatant and lesion cores, the following ELISA Kits including Mouse TNF-α ELISA Kit (EK282, MultiSciences, Hangzhou, China), Mouse IL-1β ELISA Kit (EK201B, MultiSciences, Hangzhou, China), and Mouse IL-6 ELISA Kit (EK206, MultiSciences, Hangzhou, China), were used as previously described. The absorbance was determined using a microplate reader (BioTek, Friedrichshall, Germany) at 450 nm.

**RNA isolation and qPCR**

Under low-temperature conditions, cellular lysis and RNase inhibition were achieved using Trizol reagent (Takara, Dalian, China), followed by phase separation via chloroform addition and centrifugation. Subsequently, the upper aqueous phase was collected and subjected to precipitation with isopropanol. The precipitate was then washed with ethanol, air-dried, and pure RNA was obtained. Subsequently, collected RNA were reverse-transcribed into cDNA using a reverse transcription kit HiScript II Q RT SuperMix for qPCR (R122-01, Vazyme, China). Next, qPCR reactions were performed in a 7500 real-time PCR system (Applied Biosystems, Inc., USA) according to the manufacturer’s instructions. Primer sequences were listed in Supplementary Table 3. The mRNA levels of target genes were normalized to the 18S expression. Quantification of qPCR results was performed by the 2 ^− △CT^ method.


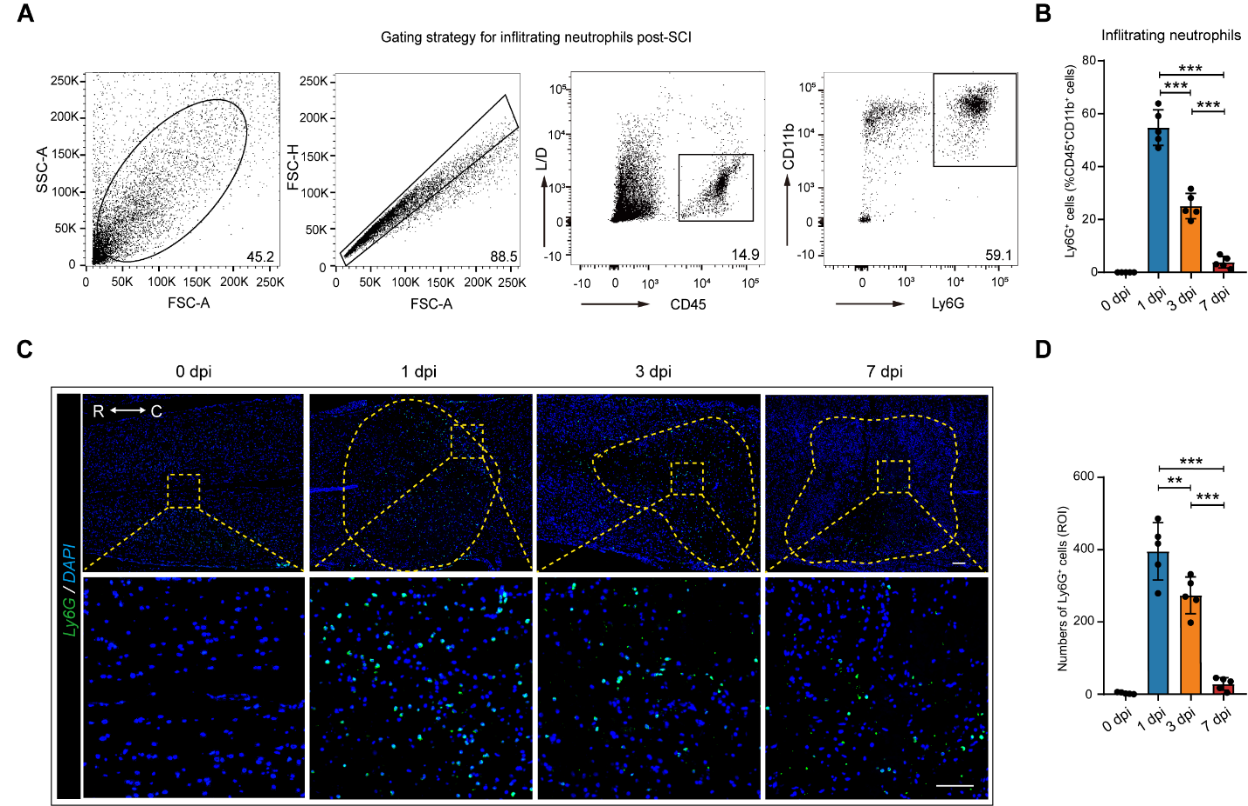


**Figure. S1｜Changes in infiltrating neutrophils post-SCI at different time points.** (**A** and **B**) Representative flow cytometry gating strategy (**A**) and statistical analysis (**B**) of infiltrating neutrophils post-SCI at different time points (n = 5). (**C** and **D**) Representative IF images (**C**) and quantification (**D**) of infiltrating neutrophils in the injured areas at 1, 3, and 7 days post-SCI (n = 5). Scale bar = 200 μm. Dashed lines indicate the lesion center and the boxed areas are shown magnified. R rostral, C caudal. All data are presented as means ± SD; ns indicates not significant, **P < 0.01, ***P < 0.001; One-way analysis respective p-values indicate of variance (ANOVA) test (**B** and **D**).


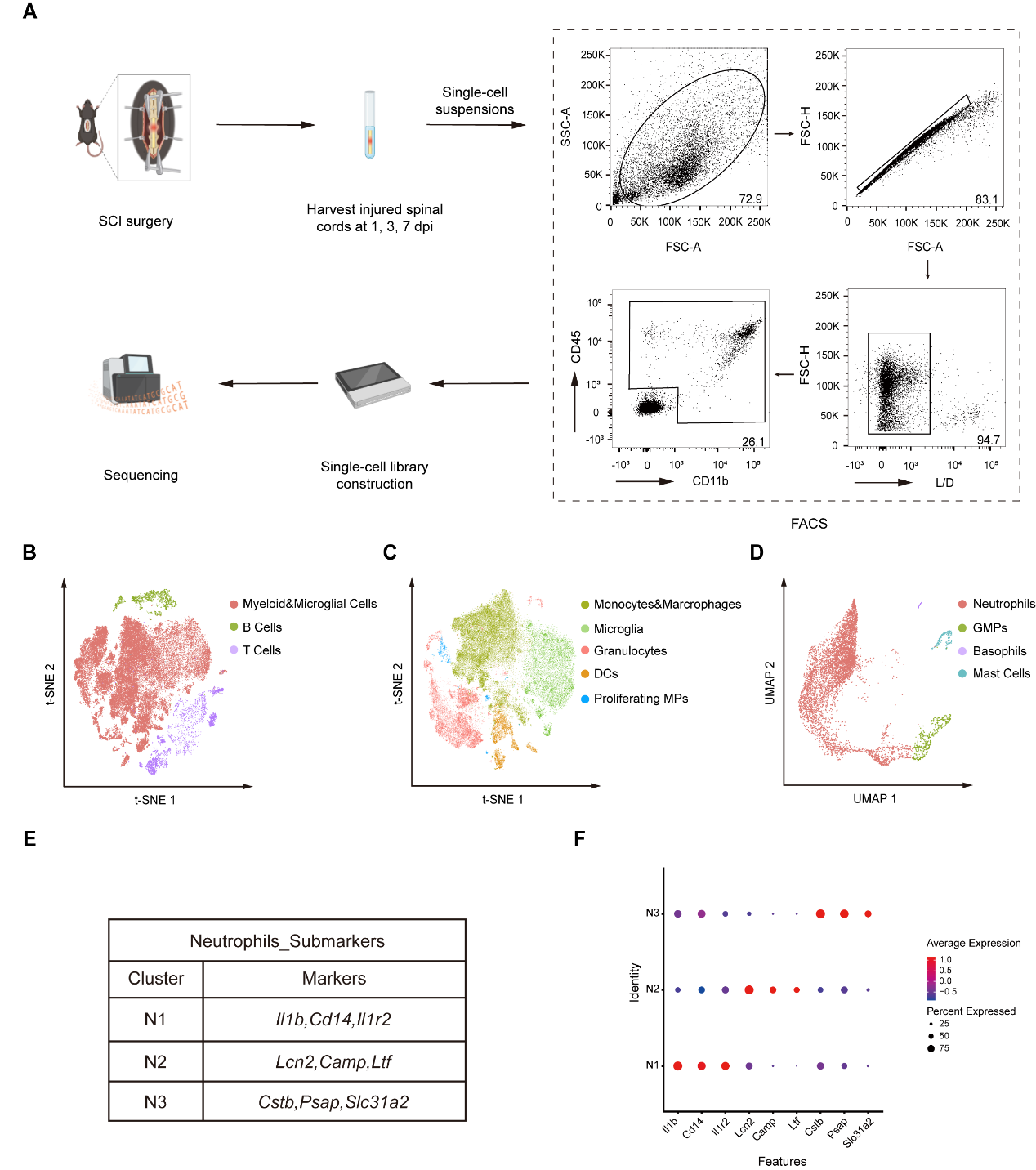


**Figure. S2｜Isolation and scRNA-seq results for immune cells post-SCI.**

(**A**) Schematic diagram of isolation and selection of infiltrating immune cells post-SCI at different time points for further scRNA-seq analysis. (**B**-**D**) t-SNE and UMAP plots showing varied clusters and population annotations. (**E**) Representative KEGG categories of upregulated DEGs in each subcluster.

**
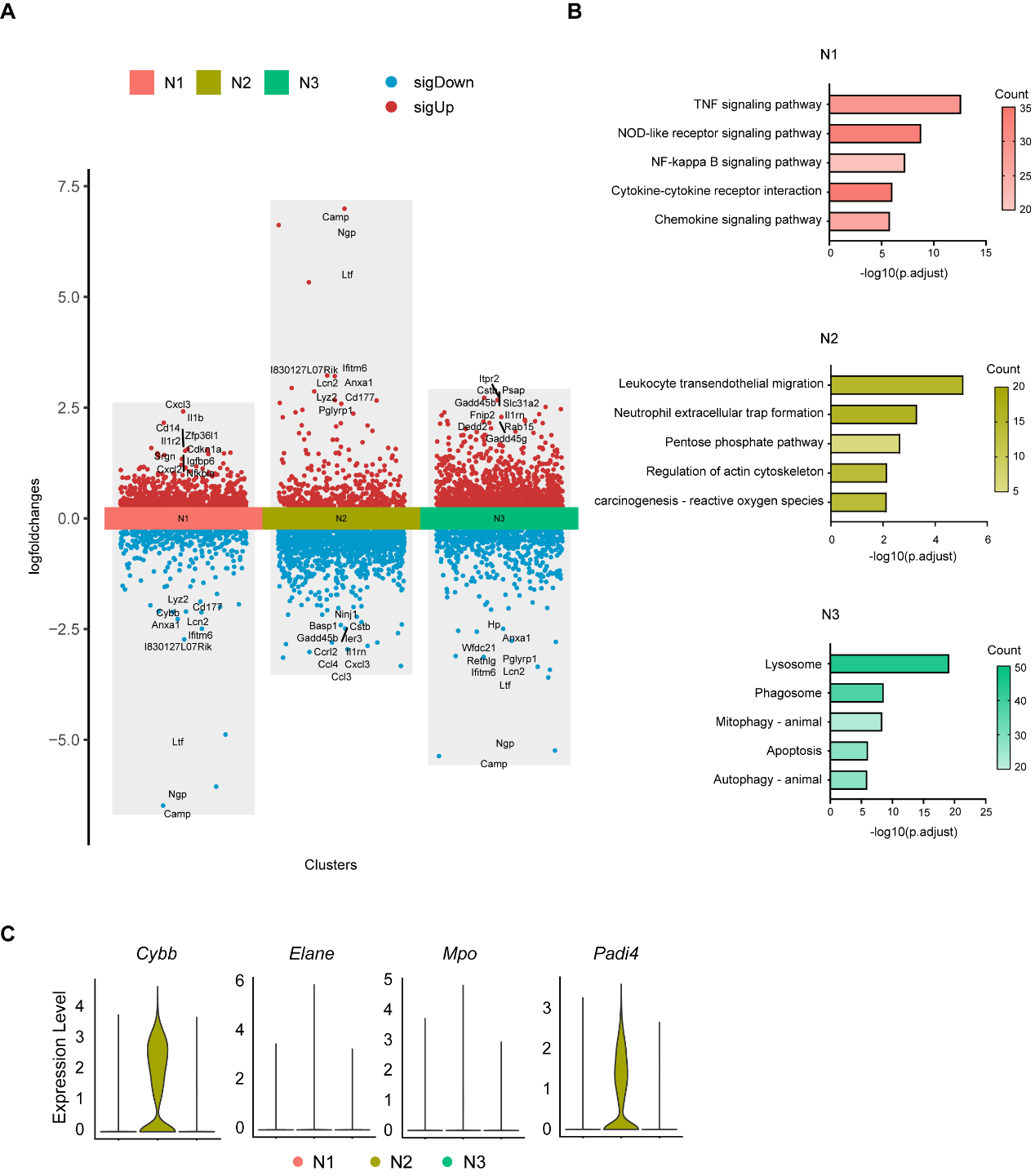
**

**Figure. S3｜Characterization of Neutrophil Subclusters in scRNA-seq data post-SCI.**

(**A**) Combined volcano graph for DEGs expression in N1-N3 subclusters. (**B**) Representative KEGG categories of upregulated DEGs in each subcluster. (**C**) Violin plot showing genes related to NET formation in individual clusters based on the scRNA-seq data.

**
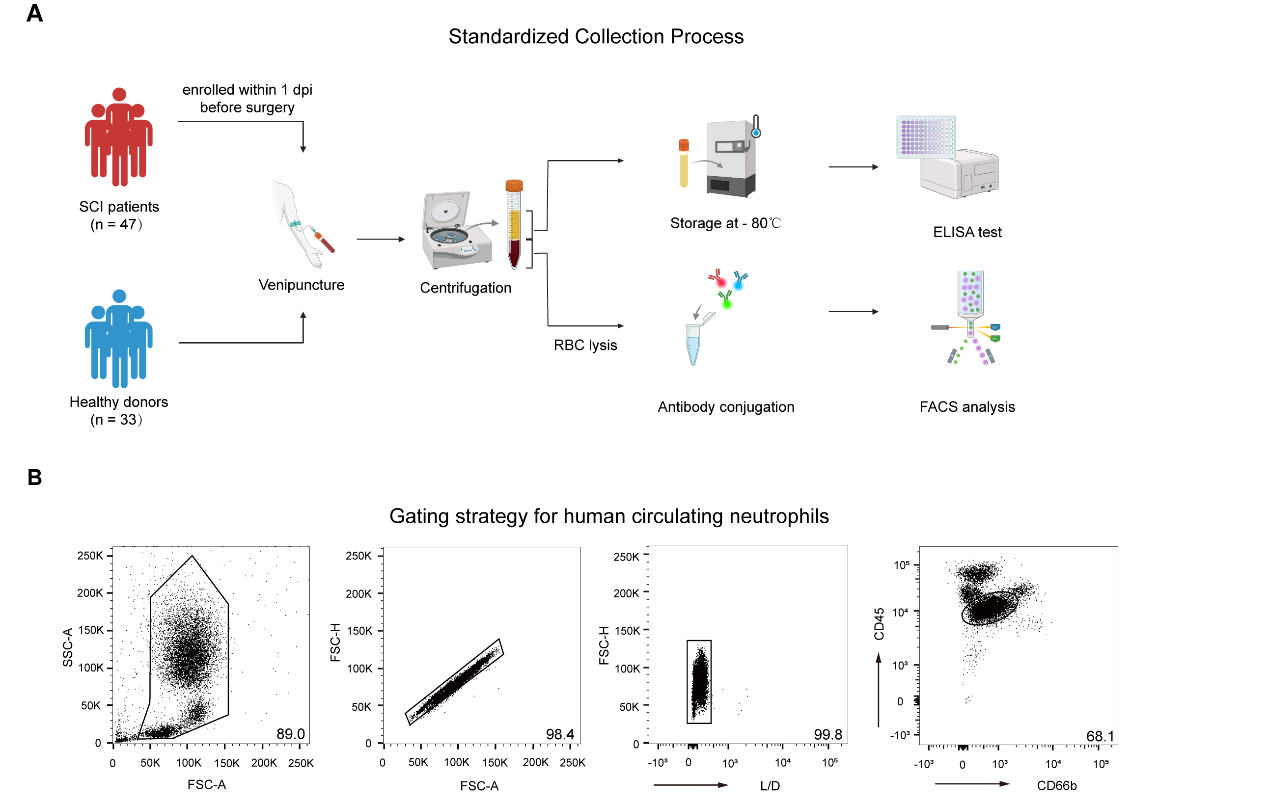
**

**Figure. S4｜Schematic of the standardized collection process and gating strategy for FACS analysis: related to Fig 2.**

(**A**) Schematic of the standardized collection process: SCI patients were enrolled within 24 hours post-injury before surgery, and venous blood samples were collected on hospital arrival. All collected samples were stored at 4°C and processed within three hours. Centrifuged plasma was stored at – 80°C for ELISA assays, and erythrocyte-lysed blood cells were marked with secondary antibodies for subsequent FACS analysis. (**B**) Representative flow cytometry gating strategy of circulating neutrophils of SCI patients (n = 33) and healthy donors (n = 33).

**
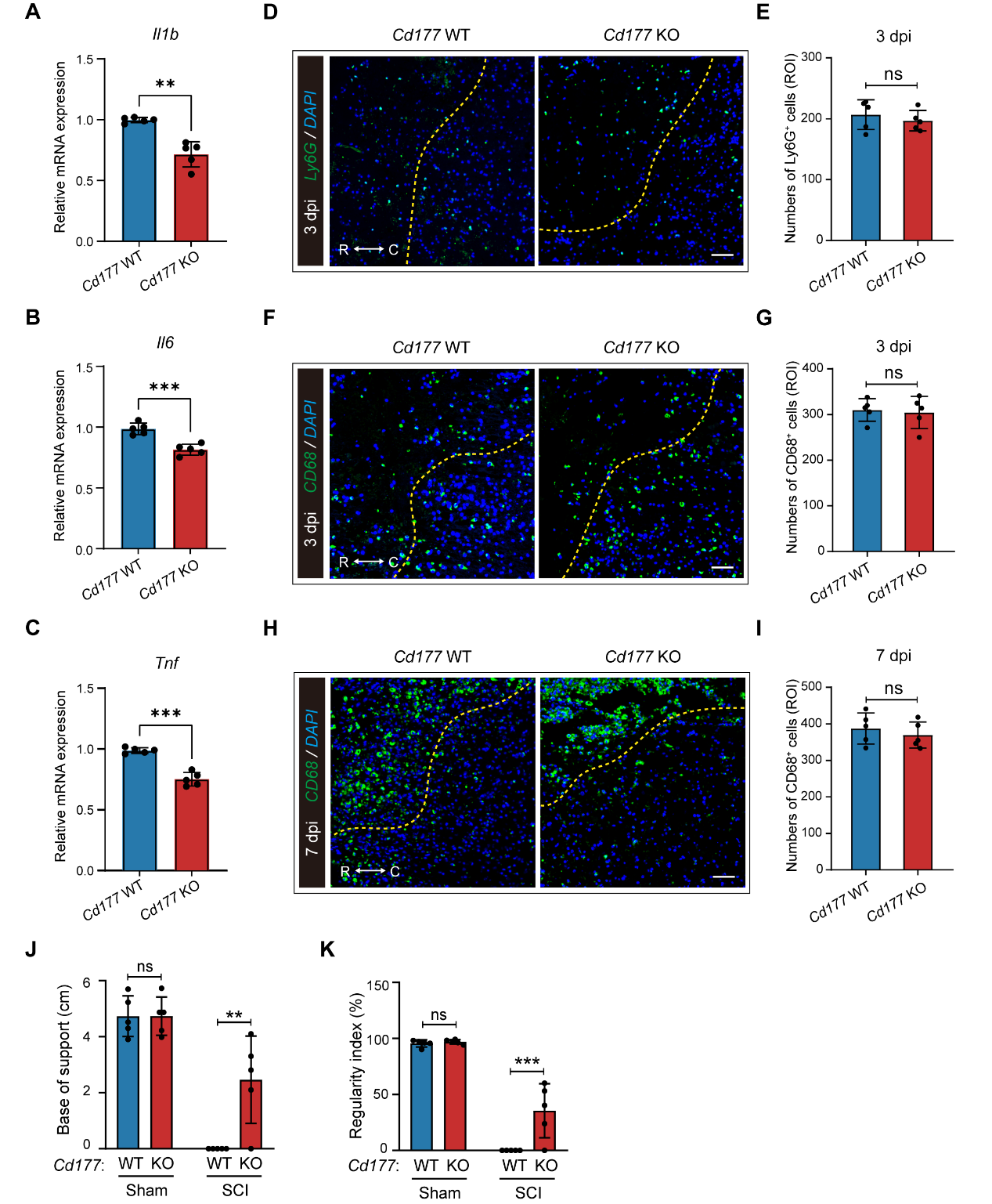
**

**Figure. S5｜Proportion of Infiltrating immune cells and Catwalk analysis in *Cd177* WT or KO mice post-SCI, related to Figure 3.**

(**A**-**C**) Quantification of pro-inflammation cytokine levels in the injured area measured by qPCR, n = 5. (**D** and **E**) Representative IF images (**D**) and quantification (**E**) of Ly6G^+^ (green) cells infiltrating the injured area at 3 dpi in the *Cd177* WT and KO mice (n = 5). (**F** and **G**) Representative IF images (**F**) and quantification (**G**) of CD68^+^ (green) cells infiltrating the injured area at 3 dpi in the *Cd177* WT and KO mice (n = 5). (**H** and **I**) Representative IF images (**H**) and quantification (**I**) of CD68^+^ (green) cells infiltrating the injured area at 7 dpi in the *Cd177* WT and KO mice (n = 5). Scale bar = 200 μm. Dashed lines indicate the injured area and the boxed areas are shown magnified. R rostral, C caudal. (**J** and **K**) Statistical analysis of Catwalk analysis results (n = 5): the base of support (BOS) reflected by the width between hind paw prints (**J**), and regularity index (**K**) reflecting the degree of coordination between front and hind paws. All data are presented as means ± SD; ns indicates not significant, **P < 0.01, ***P < 0.001; One-way ANOVA test (**J** and **K**), Unpaired two-sided Student’s t-test (**A**-**C**, **E**, **G** and **I**).

**
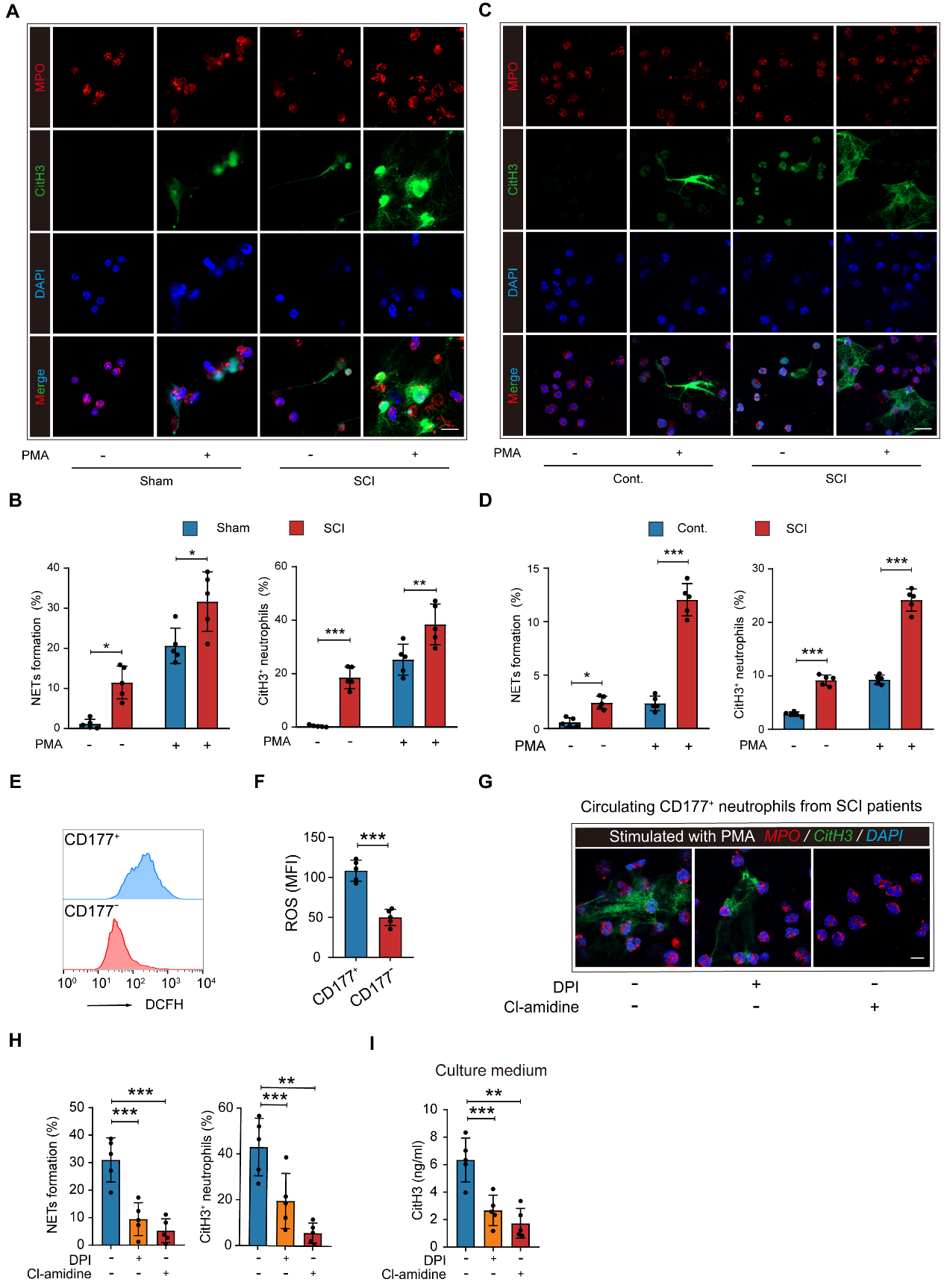
**

**Figure. S6｜NETs formation in circulating neutrophils post-SCI.**

(**A** and **B**) Representative confocal microscopy images (A) of circulating neutrophils (red) and NET release (green) from Sham or SCI mice stimulated with PMA or not. Scale bar = 20 μm. Quantification of NET formation in each group was presented in (**B**), n = 5. (**C** and **D**) Confocal microscopy images (**C**) of circulating neutrophils from SCI patients and healthy donors stimulated with PMA or not and quantification (**D**) of NET formation in each group. Neutrophils are identified by MPO (red), NETs are labeled by CitH3 (green), and nuclei are stained with DAPI (blue). Scale bar = 20 μm. (**E** and **F**) Representative histograms (**E**) and statistical analysis (**F**) measuring ROS production in circulating CD177^+^ and CD177^-^ neutrophils from SCI patients at 3 dpi (n = 5). (**G**-**I**) The inhibitory effects of ROS and PAD4 with corresponding inhibitors DPI and Cl-amidine on SCI-induced human CD177^+^ neutrophils’ NET formation were detected by IF staining (**G**) and CitH3 levels in the culture medium (**I**). Scale bar = 20 μm. Quantification of NET formation in each group was presented in (**H**), n = 5. Results in **C**, **E** and **G** were confirmed in 5 independent experiments using cells from different patients or donors. *P < 0.05, **P < 0.01, ***P < 0.001; Unpaired two-sided Student’s t-test (**F**), one-way ANOVA test (**B**, **D**, **H** and **I**).

**
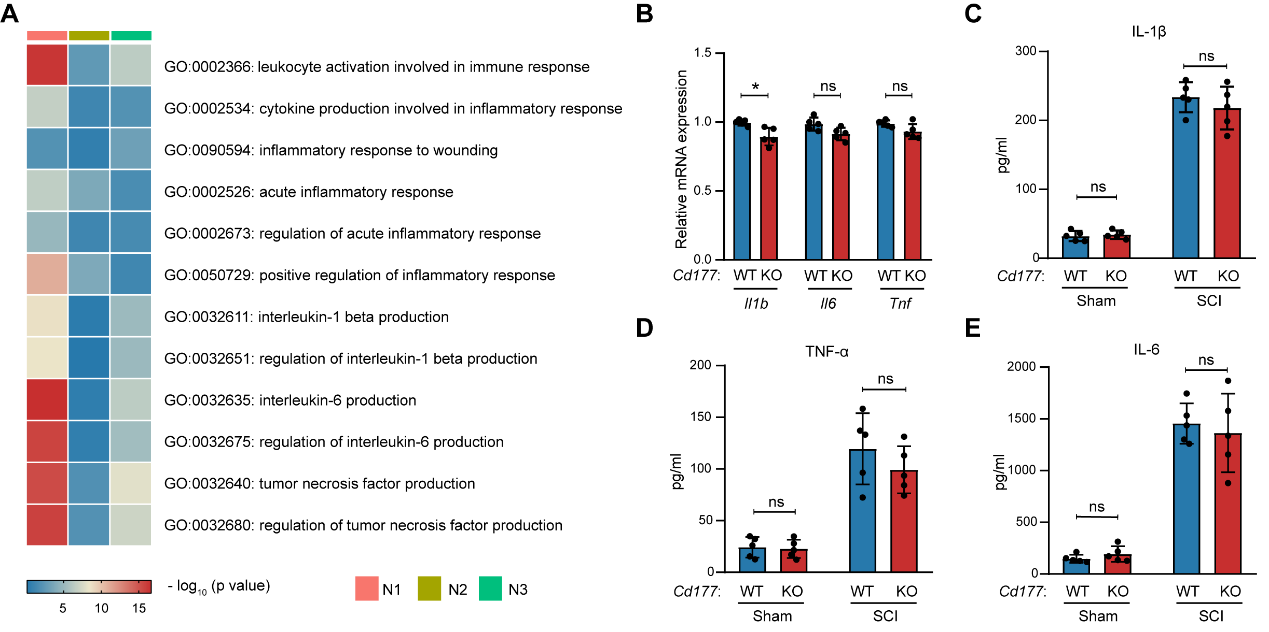
**

**Figure. S7｜ Inflammation levels of CD177^+^ neutrophils post-SCI.**

(**A**) Representative biography process (BP) categories related to inflammation were identified in GO analyses based on upregulated DEGs in each neutrophil subcluster. (**B**-**E**) Relative pro-inflammation cytokines in injured areas at 1 dpi measured through qPCR (**B**) or ELISA (**C**-**E**), n = 5. All data are presented as means ± SD; ns indicates not significant, *P < 0.05; Unpaired two-sided Student’s t-test (**B**), Two-way repeated measurement ANOVA followed by post-hoc test (**C**-**E**).

**
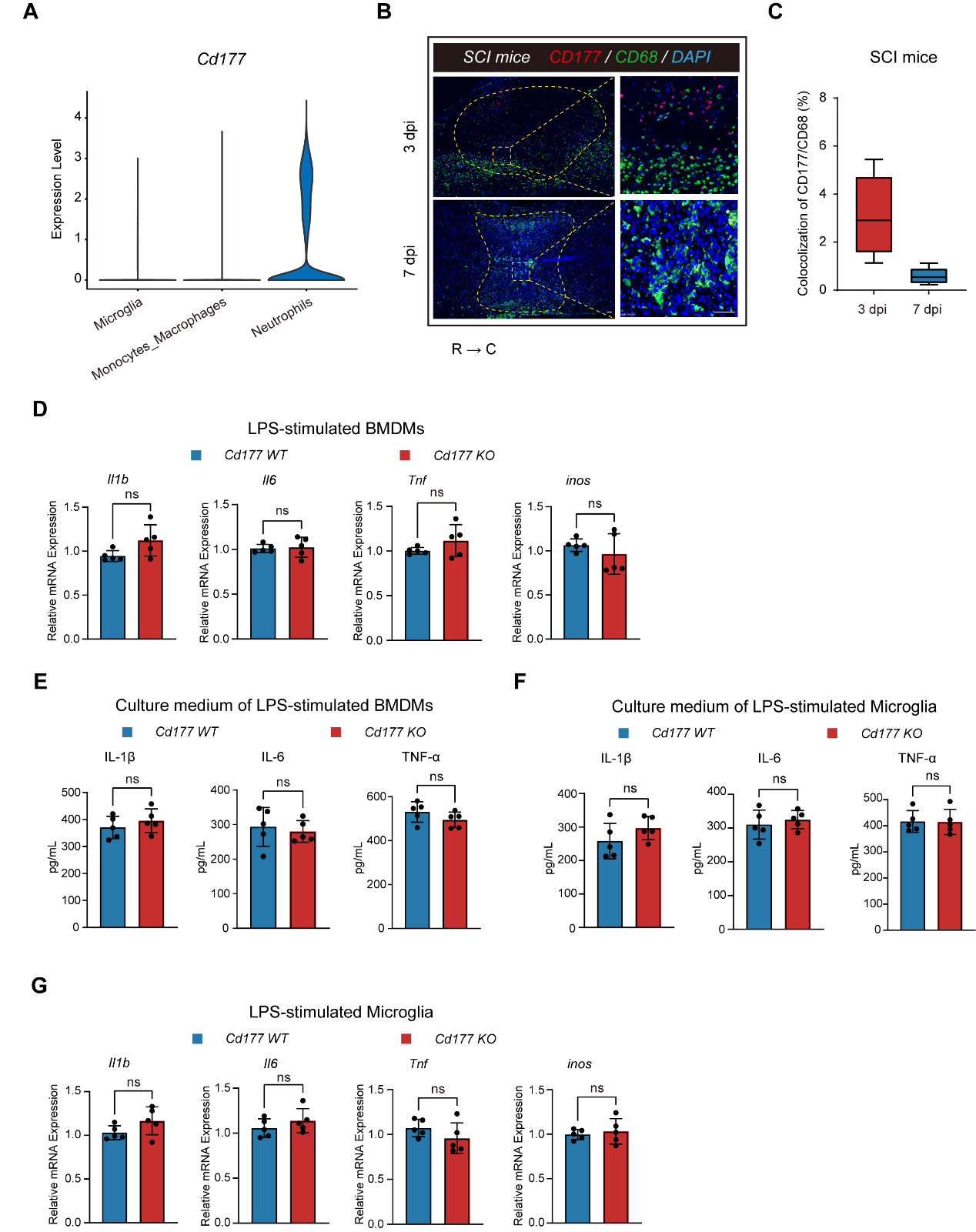
**

**Figure. S8｜The Role of CD177 in Macrophage/Microglia Expression and Inflammatory Response.**

(**A**) Violin plot showing *Cd177* expression in neutrophils, macrophages and microglia based on the scRNA-seq data. (**B** and **C**) Representative IF images (**B**) and corresponding quantification (**C**) depicting the co-localization of *CD177* (green) and CD68 (red) in the lesion sites at 3 and 7 days post-spinal cord injury (SCI) (n = 5). (**D** and **E**) Quantification of pro-inflammation cytokine levels in LPS-stimulated *Cd177* WT/KO BMDMs measured by qPCR (**D**) and ELISA (**E**), n = 5. (**F** and **G**) Quantification of pro-inflammation cytokine levels in LPS-stimulated *Cd177* WT/KO microglia measured by qPCR (**F**) and ELISA (**G**), n = 5. Scale bar = 200 μm. Dashed lines indicate the lesion center and the boxed areas are shown magnified. R rostral, C caudal. All data are presented as means ± SD; ns indicates not significant; Unpaired two-sided Student’s t-test (**D**-**G**)

**
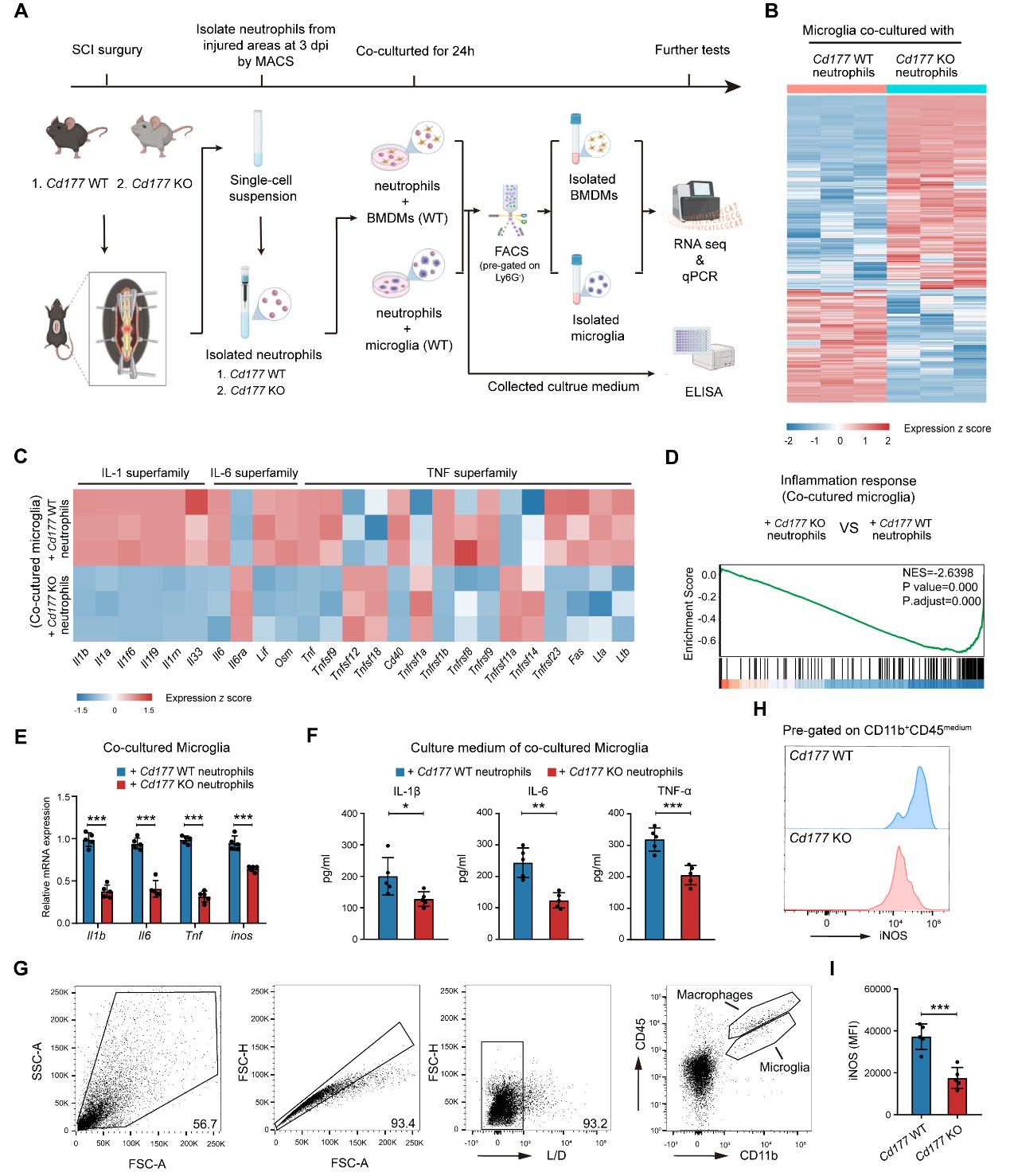
**

**Figure. S9｜*Cd177* deficiency in neutrophils inhibited pro-inflammatory polarization of BMDMs and microglia both *in vivo* and *in vitro*: related to Figure. 6.**

(**A**) Schematic diagram of isolation of infiltrating neutrophils post-SCI, co-culture system set-up, and further tests targeting BMDMs or microglia. (**B**) Heatmap of DEGs in microglia co-cultured with isolated infiltrating neutrophils from *Cd177* WT or KO mice at 3 dpi (n = 3). (**C**) Heatmap showing DEGs related to cytokine production and release in microglia co-cultured with isolated *Cd177* WT or KO neutrophils. (**D**) GSEA indicating the distribution of genes in the inflammation response pathway based on RNA-seq data. (**E** and **F**) Quantification of pro-inflammation cytokine levels in the co-culture system measured by qPCR (**E**) and ELISA (**F**), n = 5. (**H** - **I**) Representative flow cytometry analysis (**H**) and quantification (**I**) of iNOS mean fluorescence intensity (MFI) from isolated CD11b^+^CD45^medium^ cells (defined as microglia) from the injured area in *Cd177* WT or KO mice at 7 dpi (n = 5). Representative flow cytometry gating strategy of macrophages and microglia is shown in (**G**). All data are presented as means ± SD; **P < 0.01, ***P < 0.001; Unpaired two-sided Student’s t-test (**E**, **F** and **I**).

**
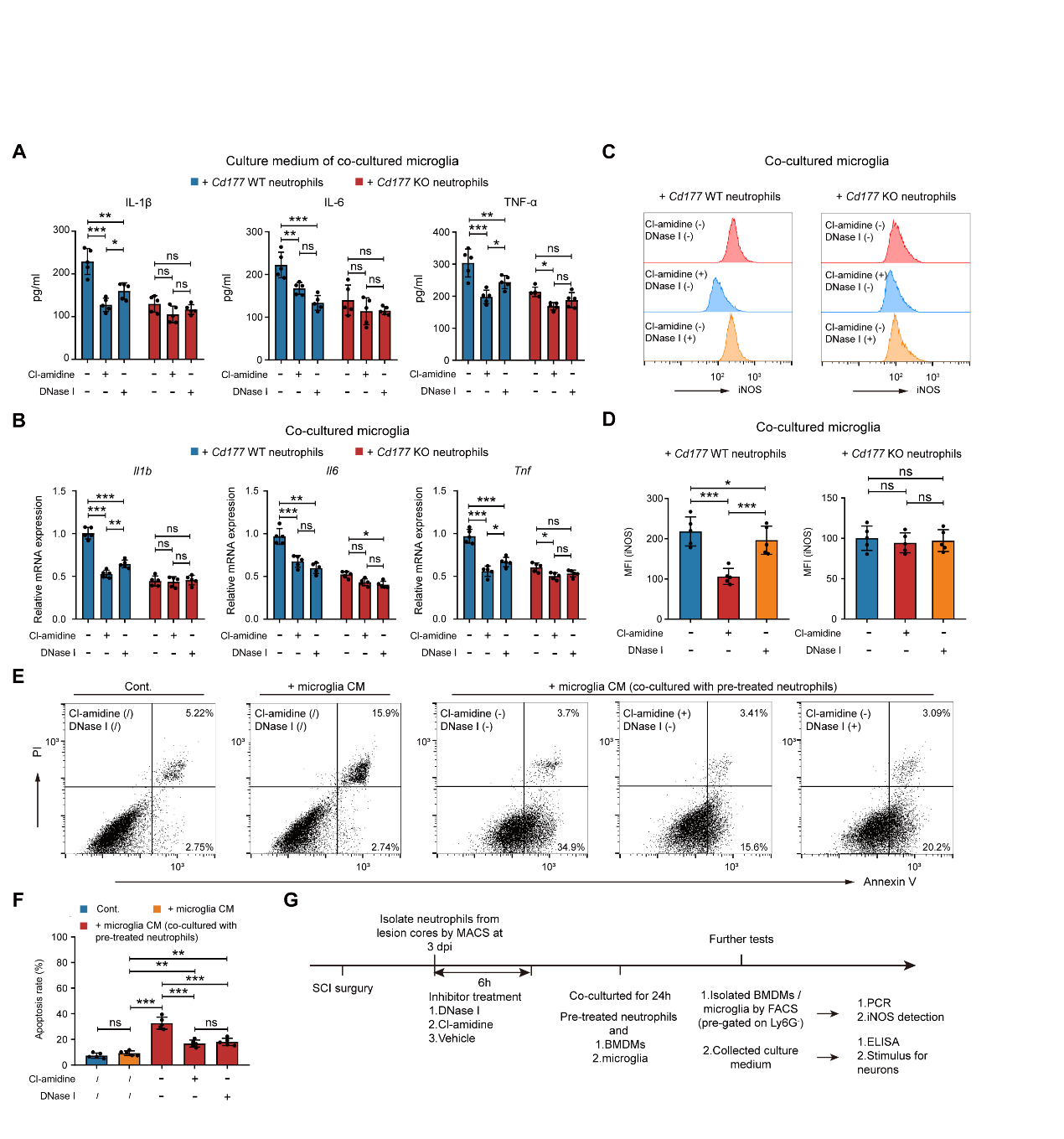
**

**Figure. S10｜Inhibition of NET formation attenuates CD177^+^ neutrophil-induced pro-inflammatory polarization of macrophages and microglia: related to Figure. 7.**

(**A** and **B**) Relative pro-inflammation cytokines of microglia were measured through ELISA (**A**) or qPCR (**B**). microglia had been co-cultured with isolated *Cd177* WT or KO neutrophils which were pre-treated with Cl-amidine, DNase I, or vehicle controls (n = 5). (**C** and **D**) Representative histograms (**C**) and statistical analysis (**D**) of the MFI show the expression of iNOS on the microglia co-cultured with isolated *Cd177* WT or KO neutrophils which were pre-treated with Cl-amidine, DNase I, or vehicle controls (n = 5). (**E** and **F**) Representative flow cytometry analysis (**E**) and quantification (**F**) of neuronal apoptosis in different groups through Annexin V-FITC/PI double staining. Neurons were treated with vehicle controls, microglia CM, or microglia CM (co-cultured with pre-treated neutrophils), n = 5. (**G**) Experimental design diagram of the co-culture system, inhibitor apply, and further tests. All data are presented as means ± SD; ns indicates not significant, *P < 0.05, **P < 0.01, ***P < 0.001; Two-way ANOVA test (**A**, **B**, **D**, and **F**).

**
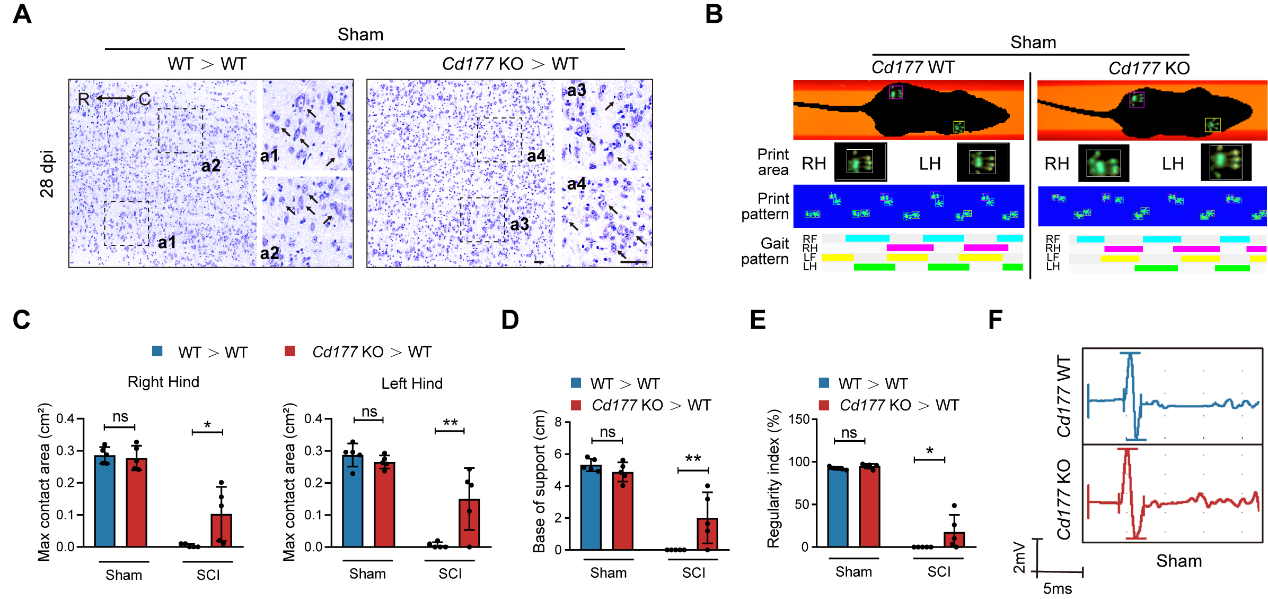
**

**Figure. S11｜Substitution of *Cd177* WT bone marrow with *Cd177* KO bone marrow eased inflammation and promoted functional recovery post-SCI: related to Figure. 8.**

(**A**) Representative photomicrographs of the Nissl-stained neurons in *Cd177* KO > WT and WT > WT groups after sham surgery at 28 dpi (n = 5). Scale bar = 200 μm. The boxed areas are shown magnified. R rostral, C caudal. (**B**) Representative images showing the maximal print area of left and right hind paws, print- and gait pattern of Catwalk analysis from *Cd177* KO > WT and WT > WT groups after sham surgery. Catwalk gait analysis was performed after the final BMS testing at 28 dpi (n = 5). (**C**, **D** and **E**) Statistical analysis of Catwalk analysis results (n = 5): evaluation of maximal contact paw print area (**C**), BOS (**D**) and regularity (**E**). (**F**) Representative images of MEP tests of hind paws in *Cd177* KO > WT and WT > WT groups after sham surgery at 28 dpi (n = 5). All data are presented as means ± SD; ns indicates not significant, *P < 0.05, **P < 0.01; One-way ANOVA test (**C**, **D** and **E**).

**
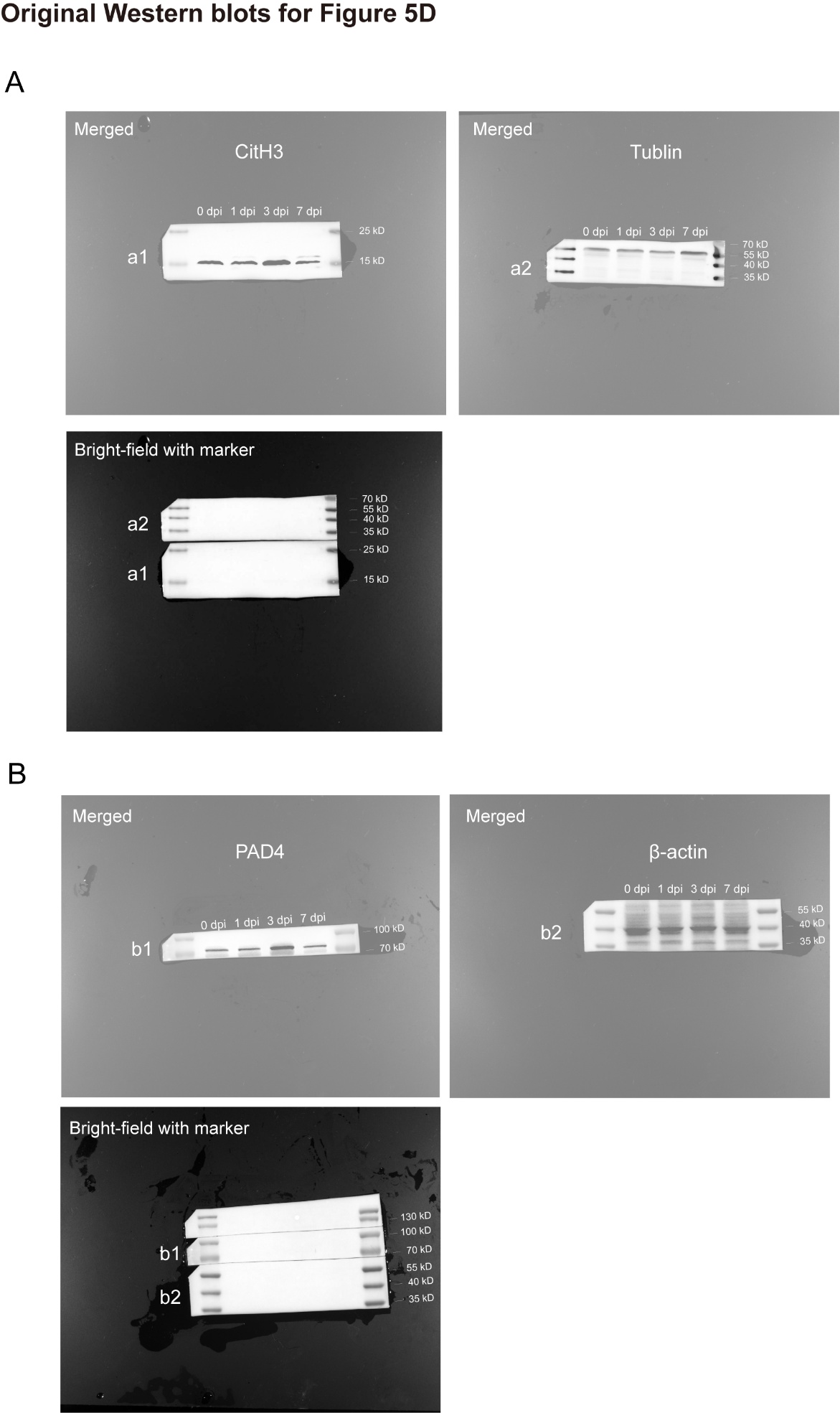
**

**Figure. S12｜Original Images of Representative Western blot images. Related to Figure. 5.**

**Table S1. Descriptive data and disease characteristics of SCI patients and healthy donors.**

|  | **SCI patients**  **(n = 47)** | **Healthy donors**  **(n = 33)** | **P-value** | **Test** |
| --- | --- | --- | --- | --- |
| **Sex (M/F)** | 29/18 | 11/12 | 0.3111 | Two-tailed Fisher's exact test |
| **Age (Years)** | 44.89 ± 11.72 | 45.79 ± 9.930 | 0.7219 | Two-tailed unpaired t test |

**Table S2. Descriptive data of SCI patients.**

| **Case** | **Sex** | **Age** | | **Neurological level of injury** | **ASIA grade** |
| --- | --- | --- | --- | --- | --- |
| 1 | M | 19 | C5, C6 | | A |
| 2 | M | 27 | C4 | | C |
| 3 | F | 33 | L1 | | B |
| 4 | M | 45 | T11 | | B |
| 5 | F | 63 | L1 | | A |
| 6 | F | 54 | C7 | | A |
| 7 | F | 60 | C7 | | A |
| 8 | M | 48 | L1 | | B |
| 9 | F | 51 | C7 | | B |
| 10 | M | 26 | L1 | | C |
| 11 | F | 20 | C7 | | A |
| 12 | F | 30 | C7 | | A |
| 13 | M | 44 | C5, C6 | | C |
| 14 | M | 42 | C3 | | A |
| 15 | M | 67 | C4 | | A |
| 16 | F | 34 | C5, C6 | | A |
| 17 | M | 54 | C7 | | B |
| 18 | M | 51 | C7 | | A |
| 19 | F | 43 | C4 | | A |
| 20 | F | 40 | C4, C5 | | A |
| 21 | M | 56 | L1 | | C |
| 22 | F | 50 | C4 | | B |
| 23 | M | 53 | T6 | | A |
| 24 | M | 38 | C7 | | A |
| 25 | F | 43 | C3 | | B |
| 26 | M | 33 | C7 | | A |
| 27 | M | 43 | T12 | | B |
| 28 | M | 47 | L1 | | A |
| 29 | F | 55 | C5, C6 | | A |
| 30 | M | 60 | C7 | | A |
| 31 | M | 59 | C3, C4 | | A |
| 32 | F | 46 | T7 | | B |
| 33 | M | 57 | T11 | | A |
| 34 | F | 38 | L1 | | B |
| 35 | M | 44 | T7 | | A |
| 36 | M | 42 | C7 | | C |
| 37 | M | 56 | T6 | | A |
| 38 | F | 55 | C3 | | B |
| 39 | M | 24 | T8 | | A |
| 40 | F | 39 | L2 | | B |
| 41 | M | 62 | C4, C5 | | A |
| 42 | M | 58 | C7 | | A |
| 43 | M | 46 | C7 | | A |
| 44 | F | 33 | T12 | | C |
| 45 | M | 36 | T12 | | A |
| 46 | M | 42 | C7 | | A |
| 47 | M | 44 | C4 | | B |

| **Table S3. Primers used in this study** | | |
| --- | --- | --- |
| **Gene** | **Sequence (5’ ﹥ 3’)** | **Species** |
| ***Il1b*** | Forward: GCAACTGTTCCTGAACTCAACT | Mouse |
|  | Reverse: ATCTTTTGGGGTCCGTCAACT |  |
| ***Il6*** | Forward: TAGTCCTTCCTACCCCAATTTCC | Mouse |
|  | Reverse: TTGGTCCTTAGCCACTCCTTC |  |
| ***Tnf*** | Forward: CAGGCGGTGCCTATGTCTC | Mouse |
|  | Reverse: CGATCACCCCGAAGTTCAGTAG |  |
| ***inos*** | Forward: GGAGTGACGGCAAACATGACT | Mouse |
|  | Reverse: TCGATGCACAACTGGGTGAAC |  |
